# Performance of quantitative measures of multimorbidity: a population-based retrospective analysis

**DOI:** 10.1186/s12889-021-11922-2

**Published:** 2021-10-18

**Authors:** Emili Vela, Montse Clèries, David Monterde, Gerard Carot-Sans, Marc Coca, Damià Valero-Bover, Jordi Piera-Jiménez, Luís García Eroles, Pol Pérez Sust

**Affiliations:** 1 Servei Català de la Salut (CatSalut), Barcelona, Spain; 2 Digitalization for the Sustainability of the Healthcare System (DS3), Barcelona, Spain; 3 Sistemes d’Informació, Institut Català de la Salut, Barcelona, Catalonia Spain; 4 Open Evidence Research Group, Universitat Oberta de Catalunya, Barcelona, Spain

**Keywords:** Multimorbidity, Chronic disease, Risk assessment, Health resources, Health planning

## Abstract

**Background:**

Multimorbidity measures are useful for resource planning, patient selection and prioritization, and factor adjustment in clinical practice, research, and benchmarking. We aimed to compare the explanatory performance of the adjusted morbidity group (GMA) index in predicting relevant healthcare outcomes with that of other quantitative measures of multimorbidity.

**Methods:**

The performance of multimorbidity measures was retrospectively assessed on anonymized records of the entire adult population of Catalonia (North-East Spain). Five quantitative measures of multimorbidity were added to a baseline model based on age, gender, and socioeconomic status: the Charlson index score, the count of chronic diseases according to three different proposals (i.e., the QOF, HCUP, and Karolinska institute), and the multimorbidity index score of the GMA tool. Outcomes included all-cause death, total and non-scheduled hospitalization, primary care and ER visits, medication use, admission to a skilled nursing facility for intermediate care, and high expenditure (time frame 2017). The analysis was performed on 10 subpopulations: all adults (i.e., aged > 17 years), people aged > 64 years, people aged > 64 years and institutionalized in a nursing home for long-term care, and people with specific diagnoses (e.g., ischemic heart disease, cirrhosis, dementia, diabetes mellitus, heart failure, chronic kidney disease, and chronic obstructive pulmonary disease). The explanatory performance was assessed using the area under the receiving operating curves (AUC-ROC) (main analysis) and three additional statistics (secondary analysis).

**Results:**

The adult population included 6,224,316 individuals. The addition of any of the multimorbidity measures to the baseline model increased the explanatory performance for all outcomes and subpopulations. All measurements performed better in the general adult population. The GMA index had higher performance and consistency across subpopulations than the rest of multimorbidity measures. The Charlson index stood out on explaining mortality, whereas measures based on exhaustive definitions of chronic diagnostic (e.g., HCUP and GMA) performed better than those using predefined lists of diagnostics (e.g., QOF or the Karolinska proposal).

**Conclusions:**

The addition of multimorbidity measures to models for explaining healthcare outcomes increase the performance. The GMA index has high performance in explaining relevant healthcare outcomes and may be useful for clinical practice, resource planning, and public health research.

**Supplementary Information:**

The online version contains supplementary material available at 10.1186/s12889-021-11922-2.

## Background

Multimorbidity is increasingly common in many countries worldwide, particularly those with higher life expectancy [1–3]. Still, most healthcare systems and therapeutic guidelines rely on disease-centred approaches, losing sight of the complexity of multimorbid patients, and hampering patient-centred approaches in clinical decision-making and healthcare planning [1]. The presence of multiple chronic conditions has been associated with lower quality of life and higher resource utilization and costs [4–7]. Hence, there is growing interest in developing measures of multimorbidity that are useful for resource planning, patient selection and prioritization, and factor adjustment in research and benchmarking [8–10].

The Charlson index, developed in the late ‘80s as a measurement of 1-year mortality risk [11], was among the first tools proposed for quantifying multimorbidity, and it is still broadly used in healthcare and research settings. Since then, various tools for assessing multimorbidity and patient complexity have been proposed, including quantitative measurements based on the count of chronic diseases (e.g., the Quality and Outcome Framework of the NHS [QOF] [8], the proposal of the Karolinska Institute for measuring chronic multimorbidity in older people [12], and the healthcare cost and utilization project [HCUP] of the US Agency for Healthcare Research and Quality [13]), and exhaustive pay tools for stratifying individuals into pre-established categories of multimorbidity (e.g., the Johns Hopkins Adjusted Clinical Groups [ACG®] [14] and the 3 M™ clinical risk groups [CRG] classification system [15]). Aside from marketed and/or nation-wide organizational tools, some authors and healthcare services nearby have explored alternative measures for summarizing the comorbidity burden and/or stratifying the population based on the health risk [16, 17].

Irrespective of the approach used, various factors challenge the development of meaningful indicators of multimorbidity. First, the concept of multimorbidity typically gravitates around chronic diseases, whereas acute conditions (e.g., hip fracture, pancreatitis) may dramatically increase patient risk and complexity [18]. Second, there is a lack of consensus regarding the criteria for identifying chronic diseases among all diagnostics [7, 19]. Finally, some of the proposed indicators (e.g., the QOF, Karolinska measure, and HCUP) are based on unweighted counts of diseases, thus losing sight of the relative contribution of each comorbidity to patient complexity [20]. While the Charlson index does provide a severity-driven weighted measure of chronic diagnostics, it is limited by the short list of diseases and severity categories considered [19].

The implementation of centralized electronic records and administrative databases for billing control in many countries has paved the way for big data strategies that allow developing population-based tools for measuring multimorbidity. The deployment of a Catalan Health Surveillance System (CHSS) in our area in 2012 prompted us to develop a population-based tool for stratifying patients according to their morbidity burden. The tool, named morbidity adjusted groups (GMA, *Grupos de Morbilidad Ajustados*), is based on the presence of chronic diseases, and it also considers recent acute diagnostic codes [21]. Like other tools, such as the ACG® and CRG® systems, the GMA is a case-mix tool that allows grouping the population according to their comorbidity burden and taking it into account when assessing outcomes of care. However, the GMA provides additional outputs at the individual level, including the number of chronic diseases, the number of organ systems affected by a chronic disease, a clinical summary label, and the multimorbidity index (i.e., a weighted measure of all diagnostics, which allow quantitative health-risk stratification at a population level) [22]. The GMA tool has shown good clinical performance—comparable with the CRGs [23, 24]—, adequate capacity to predict resource utilization in our area [25], and it has been validated in an external population using the ACG® and CRG® systems as a reference [16].

In this analysis, we assessed the performance of the multimorbidity index provided by the GMA tool in explaining health outcomes typically associated with multimorbidity and compared it to that of other quantitative measures of multimorbidity such as the Charlson index and the number of chronic diseases according to the QOF, Karolinska, and HCUP systems.

## Methods

### Population and data source

This was a retrospective cohort study based on anonymized records of the entire population of Catalonia, a North-East region in Spain with approximately 7.5 million people. The regional Health Department of Catalonia provides universal healthcare to the Catalan population through a network of 64 general hospitals, 27 psychiatry hospitals, 375 primary care centres, 91 skilled nursing facilities for intermediate care, and 130 ambulatory mental health facilities. Data were retrieved from the CHSS, which stores clinical and resource utilization information from various registries, including hospitalization, primary care visits, emergency department visits, skilled nursing facilities, palliative care, and mental health services, information on pharmacy dispensation, out-patient visits to specialists, home hospitalization, medical transportation (urgent and non-urgent), ambulatory rehabilitation, respiratory therapies, and dialysis. The source registries were originally developed for healthcare planning and invoice control to the Catalan Health Service (i.e., the public healthcare insurance in Catalonia). The registries have an automated data validation system aimed to identify inconsistencies between variables (e.g., age-diagnostic correspondence, temporal sequences, among others) and undergo external audits periodically to ensure provider payment accuracy [26]. It is estimated that approximately 20-to-25% of the Catalan population has simultaneous public and private coverage and may, therefore, use resources not reported to the CHSS. However, owing to the pharmaceutical co-payment system, nearly all patients with chronic diseases eventually visit the primary care resources (which records all chronic diagnoses) to get drug prescriptions and benefit from the pharmaceutical co-payment. All data used for the analysis were recorded in the source registries during 2017 and were retrieved in July 2019. The study protocol was approved by the Independent Ethics Committee of the IDIAP Jordi Gol (Spain) (ref. 21/042-P).

The performance of the multimorbidity tools was assessed on 10 subpopulations: all adults (i.e., aged > 17 years), and 9 subpopulations of special interest because of the expected high health risk and/or frequency of resource utilization like people aged > 64 years, people aged > 64 years and institutionalized in a nursing home for long-term care, and people with highly prevalent chronic diseases, including the following diagnose codes of the international classification of diseases (9th and 10th versions, clinical modification; ICD-9-CM and ICD-10-CM; all converted to ICD-9-CM): ischemic heart disease, cirrhosis, dementia, diabetes mellitus, heart failure, chronic kidney disease, and chronic obstructive pulmonary disease. Children and people with chronic mental health diseases were not considered as subpopulations for analysis because current measures of multimorbidity are not suited to capture the complexity of these scenarios.

### Tools for multimorbidity assessment

**Fig. 1:**
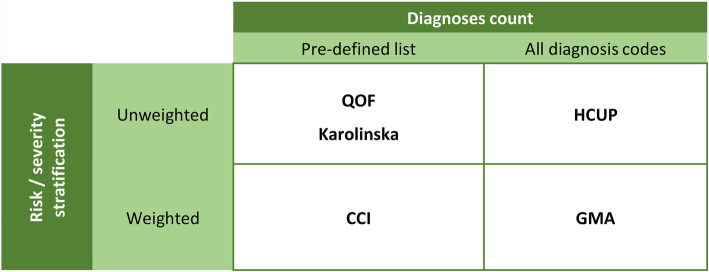
Classification of the tools for measuring multimorbidity according to the number of diagnoses included and the accountability for their severity. CCI: Charlson Comorbidity Index. GMA: adjusted morbidity groups (Spanish, *Grupos de Morbilidad Ajustada*). HCUP: healthcare cost and utilization project. QOF: Quality and Outcome Framework

Our analysis aimed to compare tools that allow summarizing the comorbidity burden using a single numeric index. In addition to the GMA tool, we selected indices frequently reported in the literature that cover different approaches regarding two features: (1) the exhaustivity in the list of diagnoses considered and (2) the weight of each diagnosis according to severity (Fig. 1). The analysis included the following five tools: the Charlson index score [11], the count of chronic diseases according to three different proposals (i.e., the QOF [8], HCUP [13], and Karolinska institute [12]), and the multimorbidity index score of the GMA tool. Briefly, the Charlson index was designed as a tool for predicting life expectancy from a list of 17 comorbidities weighted according to their 1-year risk of death. The QOF was intended as a tool for allocating healthcare resources and incentivizing care of patients with chronic diseases, and its predictive capacity for health risk indicators such as mortality has been explored [27]. The QOF tool defines multimorbidity based on the presence of more than one diagnostic from a list of 17 important chronic conditions. The HCUP measures multimorbidity by counting the number of chronic diseases among all conditions codified in the chronic condition indicator (CCI) and grouped with the clinical classification software (CCS) [28]. The HCUP defines a chronic condition based on two criteria: (a) the given disease place limitations on self-care, independent living, and social interactions, and (b) result in the need for ongoing intervention with medical products, services, and special equipment [29]. The Karolinska proposal is a clinically-driven measure of multimorbidity based on the count of chronic diseases from a list of 918 ICD-10 codes (one- to -four-digit level), which are grouped into 60 chronic disease categories to retrieve a summary measure of multimorbidity [12]. In the Karolinska proposal, chronic diseases are selected based on the following criteria, applicable to older populations: prolonged duration and either (a) left residual disability or worsening quality of life or (b) required a long period of care, treatment, or rehabilitation. The GMA tool considers all chronic diagnoses (identified using the CCI of the HCUP) present at a given time and acute diagnoses reported during the study period. The GMA index score is computed by adding the weights of each diagnosis group (defined using the CCS of the HCUP system). For instance, unlike measures based solely on diagnosis counts, the GMA multimorbidity index algorithm gives a different complexity score to patients with hypothyroidism and eczema than those with asthma and diabetes, although accounting for two chronic conditions in both cases. Supplementary file 1 provides further details on the GMA algorithm.

### Study outcomes

We investigated the contribution of each multimorbidity measure to explaining eight outcomes associated with chronic patients: all-cause death, hospitalization, non-scheduled hospitalization, number of primary care visits (including general practitioner, nurse, and social worker, either at the primary care facility, home or via teleconsultation), visits to the emergency room (ER), medication use, admission to a skilled nursing facility for intermediate care, and high expenditure [30]. All outcomes were assessed in a 1-year time frame from January 1 to December 31, 2017.

### Statistical analysis

The characteristics of the study population were described as absolute and relative frequencies and rates across all investigated outcomes. To identify individuals with high utilization rate of healthcare resources, we transformed continuous outcomes into to binary based the 95th percentile of the given variable among the overall population as cut-off. The resulting outcome definitions were as follows: admission to a skilled nursing facility for intermediate care (i.e., one or more admissions), admission to hospital (i.e., one or more admissions) and the emergency room (i.e., three or more admissions), visits to primary care services (i.e., more than 21 visits), medication use (i.e., dispensing of more than 13 drugs belonging to a different 5-digit group of the anatomic-therapeutic classification), expenditure (i.e., healthcare cost above € 4315.1). The cut-off value set for the overall study population was used for the sub-analyses on other sub-populations. Supplementary Figs. S2-S5 (Supplementary File 1) show the distribution of each study population for the continuous outcomes transformed based on the 95th percentile. To assess the performance multimorbidity measures, we built six logistic regression models for each of the investigated outcomes adjusted by age, gender, and socioeconomic status: a baseline model (i.e., age, gender, and socioeconomic status as independent variables, and all first-order interactions between them), and five models that added each multimorbidity measure to the baseline model. Multimorbidity measures were introduced into the models as continuous variables, using the indices, which were computed as described by the authors. The socioeconomic status was stratified into four categories of pharmaceutical co-payment: very low (recipient of social rescue aids), low (annual income < € 18,000), moderate (annual income € 18,000 to € 100,000), and high (annual income > € 100,000).

The performance of each model was assessed using four different statistics. For the primary analysis, we chose the area under the curve of the receiving operating characteristics (AUCROC) curve, which assesses the discrimination capacity of the model as the threshold varies and ranges from 0.5 (low discrimination capacity) to 1 (high discrimination capacity). Additionally, we conducted secondary analyses using the Akaike information criterion (AIC), pseudo-R squared (pR^2^), and the area under the precision-recall (AUC-PR). The AIC estimates the in-sample prediction error by taking into account the trade-off between the goodness of fit (overfitting) and the model simplicity (underfitting); the range of values that may take AIC depend on the study sample, with lower and higher values indicating better and poorer performance, respectively [31]. The pR^2^ assesses the goodness-of-fit and the variability explained and ranges from 0 (poor fitness of the model) to 100 (very good fitness of the model). The AUC-PR curve shows the trade-off between precision (i.e., low false-positive rate) and recall (i.e., low false-negative rate) and returns a value between 0 and 1, less biased than the ROC curve towards overestimating in outcomes with low frequency [32]. All analyses were performed using the R statistical package (version 3.6.2) [33].

## Results

### Characteristics of the study population

The analysis included 6,224,316 adult individuals (i.e., the entire adult population of Catalonia by the end of 2017) and the following subpopulations: older than 64 years (*n* = 1,472,623), older than 64 years institutionalized in a nursing home for long-term care (*n* = 67,456), ischemic heart disease (*n* = 244,311), cirrhosis (*n* = 45,126), dementia (*n* = 100,786), diabetes mellitus (*n* = 588,521), heart failure (*n* = 210,697), chronic kidney disease (*n* = 284,873), and chronic obstructive pulmonary disease (*n* = 357,989). Table 1 summarizes the main sociodemographic characteristics and rate of each study outcome for the adult population. The occurrence of most outcomes showed an increasing trend with higher age and lower socioeconomic status. Mortality was similar in the two genders; however, women tended to show higher rates of scheduled and non-scheduled hospitalization, primary care visits, ER admissions, medication use, and admissions to skilled nursing facilities. Men more frequently had expenditure below the threshold of € 4315.1. Tables S1-S9 (Supplementary file 1) summarize the characteristics of individuals included in the subpopulations.

**Table 1:** Characteristics of the study population and rate of occurrence of each of the investigated outcomes

	No. (%)	All-cause death	Hospitalization	Non-scheduled hospitalization	Primary care visits	ER utilization	Medication use	Admission to skilled nursing facility	Expenditure
Gender									
Male	3,022,978 (48.6)	1.09	8.17	4.09	4.53	3.63	4.15	0.79	6.13
Female	3,201,338 (51.4)	1.04	9.70	4.80	5.93	4.73	6.35	1.00	5.70
Age group									
18–44	2,654,178 (42.6)	0.05	4.94	2.70	1.33	3.89	0.57	0.02	1.92
45–64	2,097,515 (33.7)	0.34	6.92	2.56	3.26	2.88	3.13	0.22	5.00
65–74	729,565 (11.7)	1.19	14.61	5.80	8.50	4.68	11.48	1.07	10.56
75–84	476,422 (7.7)	3.64	22.49	11.89	19.19	7.84	21.32	3.82	17.28
> 84	266,636 (4.3)	11.90	25.34	19.89	26.17	9.73	23.50	9.27	19.64
Socioeconomic status^1^									
High	59,250 (1.0)	0.54	3.31	1.55	0.96	0.93	1.66	0.12	2.62
Moderate	1,988,779 (32)	0.65	7.01	3.05	3.10	2.50	3.30	0.42	4.21
Low	3,948,857 (63.4)	1.27	9.87	5.10	6.23	4.85	6.08	1.13	6.55
Very low	227,430 (3.7)	1.16	11.66	6.36	8.09	8.43	9.58	1.18	10.38

^1^Stratified into four categories of pharmaceutical co-payment: Very low (unemployed or recipient of social rescue aids), low (annual income < 18,000 €), moderate (annual income 18,000 to 100,000 €), and high (annual income > 100,000 €)

Categorical outcomes were transformed to binary variables using the 95th percentile of the given variable among the target population as cut-off: admission to a skilled nursing facility for intermediate care (i.e., one or more admissions), admission to emergency room (i.e., more than two admissions), visit to primary care services (i.e., more than 21 visits), medication use (i.e., dispensation of more than 13 drugs belonging to different 5-digit group of the anatomic-therapeutic classification), expenditure (.i.e., healthcare cost above 4315.1 €)

### Measures of multimorbidity

**Fig. 2:**
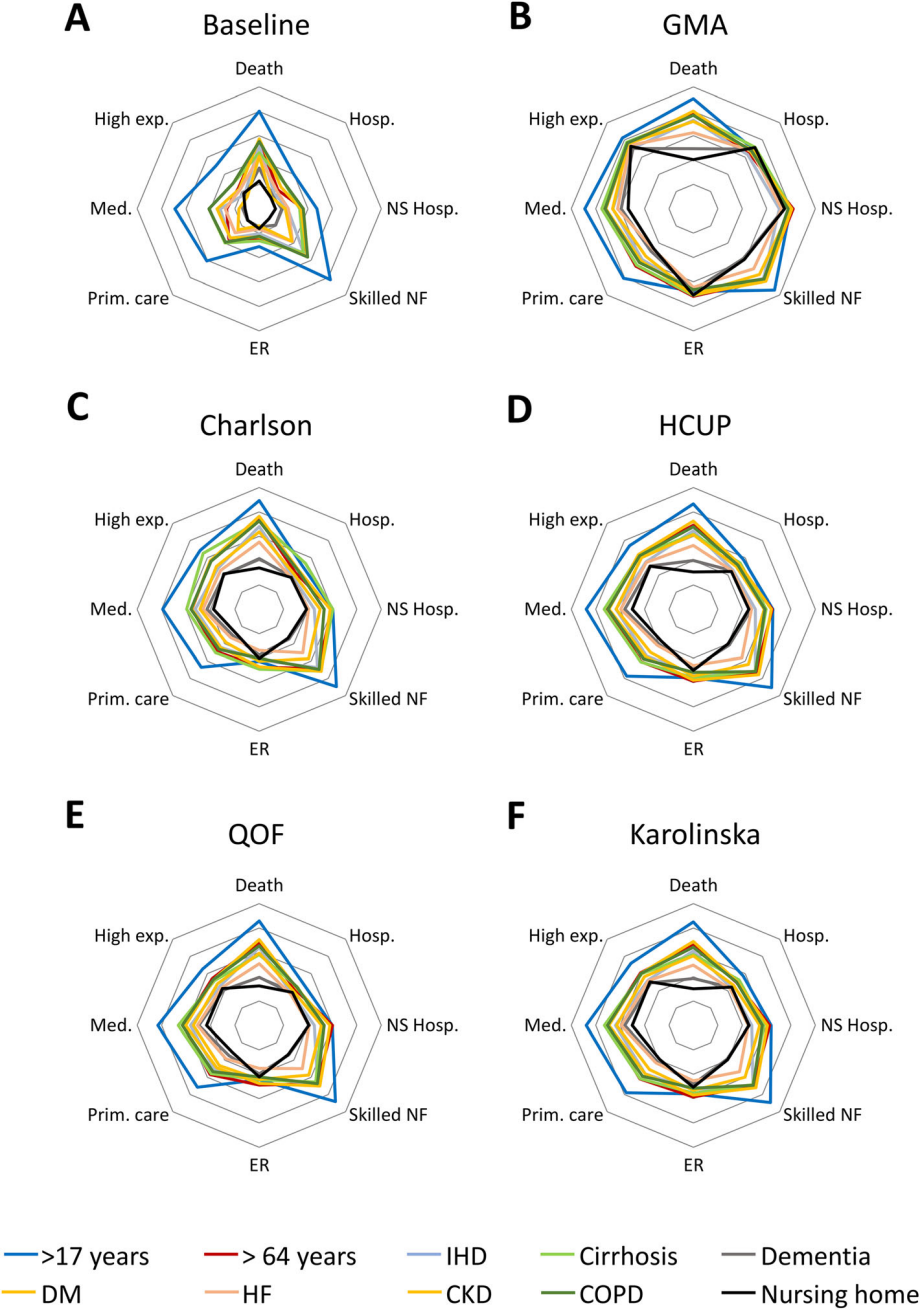
Radar plot of the performance of each multimorbidity measure in explaining health outcomes associated with chronic conditions. The plotted values of the performance of the multimorbidity measures for each outcome correspond to the area under the receiving operating curve (AUC-ROC), which ranges from 0.5 (radar centre) to 1 (external edge). **A:** reference model including age, gender, and socioeconomic status. **B**: morbidity adjusted groups (GMA) index. **C**: Charlson index. **D**: healthcare cost and utilization project (HCUP) of the US Agency for Healthcare Research and Quality. **E**: Quality and Outcome Framework of the NHS (QOF). **F**: proposal of the Karolinska Institute for measuring chronic multimorbidity in older people. For each model, the estimates are shown for ten populations: adults (aged > 17 years), people aged > 64 years, people aged > 64 years and institutionalized in a nursing home for long-term care (nursing home), and people with specific diagnoses: ischemic heart disease (IHD), cirrhosis, dementia, diabetes mellitus (DM), heart failure (HF), chronic kidney disease (CKD), and chronic obstructive pulmonary disease (COPD). The AUC values plotted in this figure are presented in Table S10 (Supplementary file 1). Tables S11-S19 present the corresponding values for the rest of the sub-populations analysed. ER: emergency room. NF: nursing facility. NS: non-scheduled

The baseline model based solely on age, gender, and socioeconomic status showed the most deficient performance in explaining the investigated outcomes in all subpopulations according to AUC-ROC estimate (Fig. 2). The poorest performance of the baseline model was consistent across all other statistics (i.e., AIC, pR^2^, and AUC-PR) (Table S10). Likewise, the addition of any multimorbidity measure to the baseline model improved the performance in explaining all investigated outcomes according to all statistics. In all models, admissions to the ER showed the lowest performance values. The GMA index (added to the baseline model) showed the highest performance in predicting all investigated outcomes. This trend was confirmed in the analyses using other statistics (Table S10). Of the other multimorbidity measures, the Charlson index score showed better performance in explaining mortality than the rest of the explored outcomes. The addition of the Karolinska and HCUP proposals for measuring multimorbidity showed better performance in explaining high use of medicines (i.e., more than 13) and primary care visits (i.e., more than 21).

Regarding the various subpopulations investigated, all models showed a trend to perform better on the general adult population and worse on people > 65 years institutionalized in nursing homes for long-term care. The GMA index showed lower variability across the investigated populations than the rest of the multimorbidity measures. This trend was confirmed when assessing performance with other statistics (Table S11-S19).

## Discussion

In this population-based, retrospective analysis of the general adult population and subpopulations of interest regarding chronic conditions, we compared the performance of various multimorbidity measures in explaining relevant healthcare outcomes associated with the management of patients with multiple chronic diseases. The baseline model of age, sex, and socioeconomic status, historically used for predicting healthcare resource utilization [34], showed the lowest performance in all investigated outcomes. Of all composites of the baseline model and multimorbidity measures, the GMA multimorbidity index performed consistently better in all outcomes, across all subpopulations, and according to the various statistical estimates used. The GMA multimorbidity index has three main advantages that may explain these results. First, like the HCUP proposal, it exhaustively considers diagnostic codes potentially associated with chronic conditions; the CCS and CCI—morbidity indicators of the HCUP, also used in the GMA proposal for identifying and classifying chronic conditions—minimizes the likelihood of duplicities. Second, although relying on chronic conditions, the GMA tool also considers recent acute diagnoses (e.g., hip fracture, pancreatitis) that may increase patient complexity and even trigger an increase in resource utilization in the mid-time horizon [18, 35]. Finally, in line with other multimorbidity measures like the Charlson index or ad hoc measures of weighted comorbidity [36], the GMA multimorbidity index rates comorbidities according to the morbidity burden or severity.

Regarding the other measures of multimorbidity investigated, the Charlson index score performed particularly well in explaining mortality. This finding is consistent with the aim of this index, which was initially developed for predicting 1-year mortality in hospitalized patients. Conversely, the two measures of multimorbidity based on diagnostics count from a short pre-selected list (i.e., the Charlson index, and the QOF) were less accurate than measures that identify chronic conditions more exhaustively (e.g., Karolinska, HCUP, GMA) in explaining outcomes associated with healthcare resource utilization such as polypharmacy and ER or primary care visits. This result could be reasonably explained by the tendency of measures based on short lists of chronic conditions towards prioritizing disabling and life-threatening diseases. While these conditions are likely to influence hard endpoints, such as institutionalization or death, they may lose sight of less severe outcomes such as increased medication use or frequency of use of healthcare resources. The definition of a chronic condition has been identified among the most critical challenges of developing multimorbidity measures, and various authors have discussed the adequate trade-off between simplicity (e.g., use of short lists) and exhaustivity of the definition approach [7, 10, 37]. In our experience, measures that consider all possible diagnostic codes (e.g., the HCUP and the GMA, both taking all diagnostic groups of the CCS) tended to perform better than those using predefined lists of diagnostics (e.g., QOF or the Karolinska proposal) in most outcomes.

Our analysis focused on multimorbidity measures that yield a numerical value (i.e., either a composite score or the number of chronic conditions) of comorbidity. While this approach excluded other complex tools such as the ACG [14] or CRG [15] systems, it allowed us quantitative comparisons of performance using statistics like the ROC-AUC. Of note, the multimorbidity index provided by the GMA tool has been previously compared with the ACG and CRG tools, showing better performance for all outcomes, except patients receiving polypharmacy [16, 23, 38]. Taken together, the high performance of the GMA tool to explain differences in a variety of outcomes broadens its applicability, including the clinical sphere (e.g., identification of patients at higher risk through a population-based approach to proactively start a closer follow-up), benchmarking between healthcare centres or areas (e.g., adjusting for multimorbidity when comparing key indicators of healthcare delivery such as re-hospitalization rates), and healthcare resource allocation (e.g., anticipating resource needs or prioritizing in case of resource shortage [39]).

Our analysis was limited by the use of administrative databases, which precluded us from investigating non-recorded outcomes such as quality of life or physical function. In fact, population-based multimorbidity tools, such as those included in our analysis, are typically designed for healthcare planning and resource allocation. Hence, although we have not tested the performance of the GMA for explaining differences in physical or quality of life decline at the individual level, other indexes are expected to perform better on these outcomes [20, 36, 40]. Furthermore, the retrospective design provided an explanatory approach of healthcare outcomes; future studies shall assess the predictive capacity of these measures prospectively. On the other hand, the analysis was strengthened by the consistency of the main results across the various statistical estimates and subpopulations and the population-based approach, which allowed us to test the multimorbidity measures on a study population of over six million people. Of note, although the source datasets collect only resources afforded by the public insurance system, nearly all people with chronic diseases eventually visit the primary care resources to benefit from the pharmaceutical co-payment. Hence, the CHSS is unlikely to miss information on chronic diagnoses. The frequency of visits to the specialist, which is more likely to be biased in people with double healthcare coverage, was not included among study outcomes.

## Conclusions

Our results show that the addition of a quantitative measure of multimorbidity to variables considered traditionally explanatory of healthcare outcomes—such as age, gender, and socioeconomic status—increases the performance of the model in explaining these outcomes. In our analysis, the GMA multimorbidity index performed better than other quantitative measures of multimorbidity in explaining relevant outcomes like all-cause death, total and non-scheduled hospitalization, primary care and ER visits, medication use, admission to a skilled nursing facility for intermediate care, and high expenditure. These findings provide policymakers and medical directors with strong evidence on the use of multimorbidity tools for clinical practice, resource planning, and public health researchers with useful insights for health risk stratification.

## Supplementary Information

**Additional file 1:** Supplementary methods (algorithm description) and supplementary results (**Tables S1-S19** and **Figures S1-S5**).

## Data Availability

The datasets generated and/or analysed during the current study are not publicly accessible but are available from the corresponding author upon reasonable request.

## Abbreviations

ACG: adjusted clinical groups
AIC: Akaike information criterion
AUC: area under the curve
CCI: chronic condition indicator
CCS: clinical classification software
CHSS: Catalan health surveillance system
CRG: clinical risk groups
ER: emergency room
GMA: adjusted morbidity groups (from Spanish, *Grupos de Morbilidad Ajustada*)
HCUP: healthcare cost and utilization project
ICD: international classification of diseases
PR: precision recall
QOF: quality and outcome framework
ROC: receiving operating characteristics
