# Supplementary material for: Performance of quantitative measures of multimorbidity: a population-based retrospective analysis
Source: BMC Public Health. 2021 Oct 18;21:1881. doi: 10.1186/s12889-021-11922-2 (PMC8524794; doi:10.1186/s12889-021-11922-2)

Performance of five tools for measuring multimorbidity in explaining health outcomes in the general population; a cross-sectional, population-based analysis

Emili Vela,[1,2] Montse Clèries,[1,2] David Monterde,[1,2,3] Gerard Carot-Sans,[1,2] Marc Coca,[1,2] Damià Valero,[1,2] Jordi Piera-Jiménez,[1,2,3] Luís García Eroles,[1,2] and Pol Pérez Sust.[1]

1. Servei Català de la Salut, Barcelona, Spain.
2. Digitalization for the Sustainability of the Healthcare System (DS3), Sistema de Salut de Catalunya, Barcelona, Spain.
3. Sistemes d'Informació, Institut Català de la Salut, Barcelona, Catalonia, Spain
4. Open Evidence Research Group, Universitat Oberta de Catalunya, Barcelona, Spain

Supplementary file 1

1 CONTENTS

2 Supplementary Materials 3
2.1 Algorithm for health risk stratification according to the adjusted morbidity groups 3
Algorithm Input 3
Data processing 4
Output 5
References 5
3 Supplementary results 6
3.1 Supplementary Tables 6
Characteristics of study subpopulations. 6
Performance of multimorbidity measurements in all populations 16
3.2 Supplementary Figures 37

2 SUPPLEMENTARY MATERIALS

2.1 ALGORITHM FOR HEALTH RISK STRATIFICATION ACCORDING TO THE ADJUSTED MORBIDITY GROUPS

Figure S1. Algorithm overview of the adjusted morbidity groups (GMA) tool

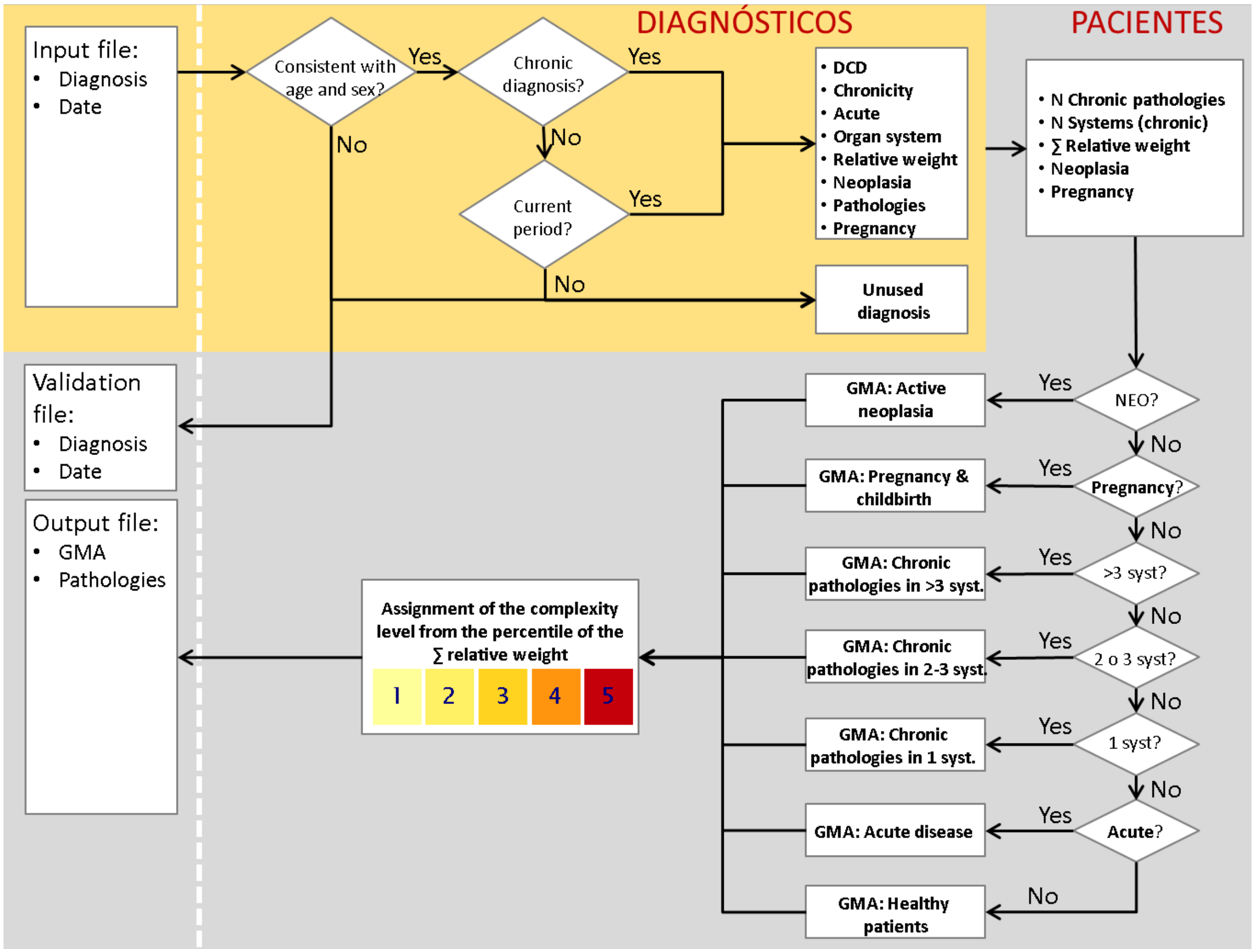

ALGORITHM INPUT

The input file of the model contains essential personal information of the individual (i.e., identification number, birth date, and sex) and a list of his/her diagnoses (and/or relevant health conditions). Each record in the input file corresponds to a diagnostic/health condition and includes the following variables:

- **Type of diagnostic classification used.** The GMA tool has been designed to accept the following international codifications: ICD-9 CM, ICD-10, ICD-10-CM, CIAP-1, and CIAP-2. The system can handle different classifications simultaneously (e.g., for a given individual, illnesses diagnosed in the primary care and hospital settings may be recorded using different classification systems).
- **Code of the health condition.**
- **Date of diagnosis**.

DATA PROCESSING

Before processing input records, the algorithm assesses the consistency of the diagnostic code with age and gender (inconsistencies are recorded in a validation file that can be reviewed later) and validates the diagnostic date (chronic conditions diagnosed after the end of the analyzed period and acute conditions diagnosed before the analyzed period are excluded). Then, the algorithm generates a table with the following information regarding the diagnostic or health condition:

- **Diagnostic code**.
- **Identification of diagnostic code group**. Instead of considering all possible diagnostic codes individually (diagnostic classification systems may exceed 90,000 codes depending on the exhaustivity level), the GMA tool uses the diagnostic code groups (DCG), which groups all codes associated with a given disease.
- **Identification of chronicity**. Diagnoses are identified as a chronic condition based on the criteria of the healthcare cost and utilization project (HCUP) of the US Agency for Healthcare Research and Quality,[1] which considers all diseases with the following characteristics: (a) the given disease place limitations on self-care, independent living, and social interactions, and (b) result in the need for ongoing intervention with medical products, services, and special equipment.[2]
- **Identification of acute pathology**. Individuals with any DCG associated with an acute disease with a diagnosis date within the analysis period.
- **Identification of neoplasia**. Individuals with any DCG associated with any neoplasia code and diagnosis date within the analysis period or a year before.
- **Identification of pregnancy and/or childbirth**. Individuals with any DCG associated with pregnancy and/or delivery within the analysis period.
- **Identification of the affected organ system**. The algorithm discriminates between chronic conditions that affect different systems and those that affect the same system. Each DCG is assigned to a system according to the criteria of the international classification of diseases: infections, neoplasms, digestive system, circulatory system, etc.
- **Weighted complexity of the DCG**. The complexity level of each DCG is determined based on three relevant information blocks corresponding to individuals with the given DCG: (1) mortality, (2) healthcare needs (i.e., number of scheduled and unscheduled hospital admissions and primary care visits), and (3) number of prescribed drugs. The weight values have been obtained by modeling DCG and outcome data from the catchment population of the Catalan Healthcare System (7.5 million people; data collected in 2011).
- **Relevant pathology label**. The algorithm identifies the presence of relevant diseases among a pre-defined list of 80 DCGs. Neoplasms are classified as active or previous.

Individuals are first assigned to a given morbidity group code by checking the presence of the presence of the following conditions sequentially:

- Active neoplasia (code 40).
- Pregnancy and/or childbirth-related pathology (code 20).
- 4 ≥ systems affected by a chronic condition (code 33).
- 2-to-3 systems affected by a chronic condition (code 32).
- 1 system affected by a chronic condition (code 31).
- Acute illness (code 10).
- Healthy (code 00).

The morbidity group code is complemented with a 4-point complexity score, assigned based on cutoff points corresponding to the 40th, 70th, 85th, and 95th percentiles of the complexity distribution in the entire population.

OUTPUT

The algorithm releases the following summary information regarding the morbidity burden:

- **The morbidity group**. Combination of the morbidity group code and group complexity score.
- **Clinical label**. List of relevant diseases.
- **Morbidity index**. Weighted complexity of DCGs at the individual level. Since the morbidity index is a continuous variable, it can be used to allocate each individual into one of the four risk groups established according to the distribution of the general population: healthy (up to 50th percentile), low-risk group (50th to 85th percentile), moderate-risk group (85th to 95th percentile), and high-risk group (above 95th percentile).

REFERENCES

1. Agency for Healthcare Research and Quality. Healthcare Cost and Utilization Project (HCUP). Available at: https://www.ahrq.gov/data/hcup/index.html. Accessed December 3, 2020.

2. Perrin EC, Newacheck P, Pless IB, *et al.* Issues involved in the definition and classification of chronic health conditions. *Pediatrics* 1993; **91**: 787–93.

3 SUPPLEMENTARY RESULTS

3.1 SUPPLEMENTARY TABLES

CHARACTERISTICS OF STUDY SUBPOPULATIONS.

Tables S1 to S9 summarize the demographic, socioeconomic, and clinical characteristics of individuals included in each of the sub-populations, as well as the occurrence of investigated outcomes. The following subpopulations are described in tables S1 to S9, respectively: general population aged >65 years, ischemic heart disease, cirrhosis, dementia, diabetes, heart failure, chronic kidney disease, chronic obstructive pulmonary disease, older than 64 years institutionalized in a nursing home for long-term care.

Table S1. Characteristics of the study population and rate of occurrence of each of the investigated outcomes. Subgroup **>65 years**. Outcome results are presented as percentages.

	No.	All-cause death	Hospitalization	Non-scheduled hospitalization	Primary care visits	ER utilization	Medication use	Admission to skilled nursing facility	Expenditure
**Gender**									
Male	633,060	4.32	20.98	11.75	14.62	7.16	15.29	3.25	16.20
Female	839,563	3.62	17.69	9.25	15.56	6.21	18.01	3.59	13.01
**Age group**									
65-69	387,361	0.98	12.85	4.95	6.75	4.12	9.32	0.80	9.22
70-74	342,204	1.43	16.61	6.77	10.48	5.31	13.93	1.39	12.08
75-79	241,647	2.55	21.16	9.84	16.16	7.03	19.38	2.71	15.90
80-84	234,775	4.77	23.87	13.99	22.32	8.68	23.31	4.97	18.71
85-89	164,848	8.64	25.35	18.60	26.19	9.93	24.68	8.06	20.21
90-94	77,991	15.25	25.81	22.09	26.82	9.87	22.65	11.05	19.57
>95	23,797	23.57	23.77	21.65	23.94	7.91	18.12	11.79	15.88
**Socioeconomic status**[1]									
High	11,987	2.09	6.74	3.53	3.32	1.69	5.81	0.50	5.74
Moderate	437,653	2.49	14.77	7.07	9.27	4.46	11.36	1.73	10.50
Low	982,899	4.56	21.13	11.79	17.81	7.52	19.19	4.19	16.10

Very low	40,084	4.39	20.60	12.03	18.01	9.50	22.39	4.67	17.08

[1]Stratified into four categories of pharmaceutical copayment: Very low (unemployed or recipient of social rescue aids), low (annual income < 18,000 €), moderate (annual income 18,000 to 100,000 €), and high (annual income >100,000 €).

Categorical outcomes were transformed to binary variables using the 95th percentile of the given variable among the target population as cutoff: hospital admission (i.e., one or more admissions), non-scheduled hospitalization (i.e., one or more admissions), admission to a skilled nursing facility for intermediate care (i.e., one or more admissions), admission to emergency room (i.e., more than two admissions), visit to primary care services (i.e., more than 21 visits), medication use (i.e., dispensation of more than 13 drugs belonging to different 5-digit group of the anatomic-therapeutic classification), expenditure (.i.e., healthcare cost above 4,315.1 €)

Table S2. Characteristics of the study population and rate of occurrence of each of the investigated outcomes. Subgroup **ischemic heart disease**. Outcome results are presented as percentages.

	No.	All-cause death	Hospitalization	Non-scheduled hospitalization	Primary care visits	ER utilization	Medication use	Admission to skilled nursing facility	Expenditure
**Gender**									
Male	157,469	5.87	30.42	20.53	20.91	11.88	28.68	4.25	28.15
Female	86,842	7.32	31.36	22.35	29.37	14.20	40.28	7.08	28.44
**Age group**									
0-14	654	0.31	11.77	4.74	6.42	10.86	0.76	0.00	9.33
15-24	553	0.18	11.03	5.24	4.16	11.75	1.45	0.00	7.41
25-34	916	0.22	19.98	15.39	8.62	15.07	3.82	0.11	14.30
35-39	1,259	0.40	22.48	15.65	6.99	13.50	6.35	0.24	19.62
40-44	2,859	0.94	22.07	16.19	10.00	12.00	9.13	0.45	20.08
45-49	5,773	0.87	22.40	15.57	9.44	10.25	11.43	0.47	21.95
50-54	10,662	1.15	22.77	15.48	10.51	9.57	14.52	0.66	22.87
55-59	16,563	1.64	23.93	15.52	11.29	8.71	17.41	0.89	22.95
60-64	22,393	1.96	24.05	14.31	13.06	8.40	21.75	1.26	23.00
65-69	30,068	2.55	27.02	15.90	16.02	10.13	28.18	1.87	25.54
70-74	35,578	3.39	29.73	17.72	21.54	11.43	34.47	2.85	28.01
75-79	32,290	5.32	33.86	21.51	28.06	13.83	40.83	4.74	31.29
80-84	37,679	8.36	36.62	26.19	34.52	15.80	43.76	7.85	33.59
85-89	29,233	13.29	37.60	30.45	36.71	16.96	43.51	11.84	33.20
90-94	13,929	20.39	36.38	32.50	35.68	16.66	38.97	15.28	30.43
>95	3,902	28.52	34.01	31.37	30.60	12.97	31.83	16.48	24.81
**Socioeconomic status**[1]									
High	1,940	2.94	14.07	9.12	5.72	3.81	12.27	0.62	14.23
Moderate	68,197	4.24	25.48	16.31	16.12	8.79	23.97	2.83	22.37
Low	165,332	7.32	33.00	23.18	27.23	14.13	36.27	6.30	30.52
Very low	8,842	6.15	33.00	24.01	26.05	18.15	40.70	5.41	34.23

[1]Stratified into four categories of pharmaceutical copayment: Very low (unemployed or recipient of social rescue aids), low (annual income < 18,000 €), moderate (annual income 18,000 to 100,000 €), and high (annual income >100,000 €).

Categorical outcomes were transformed to binary variables using the 95th percentile of the given variable among the target population as cutoff: hospital admission (i.e., one or more admissions), non-scheduled hospitalization (i.e., one or more admissions), admission to a skilled nursing facility for intermediate care (i.e., one or more admissions), admission to emergency room (i.e., more than two admissions), visit to primary care services (i.e., more than 21 visits), medication use (i.e., dispensation of more than 13 drugs belonging to different 5-digit group of the anatomic-therapeutic classification), expenditure (.i.e., healthcare cost above 4,315.1 €)

Table S3. Characteristics of the study population and rate of occurrence of each of the investigated outcomes. Subgroup **cirrhosis**. Outcome results are presented as percentages.

	No.	All-cause death	Hospitalization	Non-scheduled hospitalization	Primary care visits	ER utilization	Medication use	Admission to skilled nursing facility	Expenditure
**Gender**									
Male	26,798	7.01	29.91	20.08	13.58	12.71	16.66	4.55	29.19
Female	18,328	4.87	25.03	15.38	16.31	11.62	22.77	4.54	23.75
**Age group**									
0-14	372	0.00	11.02	6.18	4.30	8.60	0.81	0.00	8.60
15-24	329	0.00	7.60	4.26	3.34	7.90	2.43	0.00	6.99
25-34	890	0.45	12.92	6.74	2.81	9.21	2.02	0.34	7.64
35-39	1,074	0.74	12.57	8.57	4.56	8.29	2.98	0.47	9.50
40-44	1,939	1.29	12.48	8.41	5.36	8.66	4.90	0.83	13.51
45-49	3,056	2.68	18.49	12.53	7.40	10.31	7.79	1.41	19.67
50-54	4,764	3.27	22.08	14.34	8.35	10.52	10.79	1.66	23.95
55-59	5,815	3.77	24.90	15.72	9.41	9.94	13.07	2.27	26.07
60-64	5,774	4.99	27.88	17.16	11.57	10.81	16.68	3.50	27.21
65-69	5,875	5.48	29.72	17.57	14.50	11.49	20.97	4.22	28.31
70-74	5,601	6.70	32.05	19.07	17.80	13.27	26.01	4.73	29.99
75-79	3,909	9.54	38.09	25.04	23.94	16.83	33.33	7.52	36.15
80-84	3,270	12.81	40.61	29.05	30.83	17.83	36.39	11.44	37.98
85-89	1,782	19.25	42.26	34.23	32.55	20.09	34.62	15.32	36.20
90-94	574	22.47	38.85	35.37	31.88	15.33	31.53	17.07	33.10
>95	102	27.45	34.31	33.33	27.45	13.73	25.49	18.63	23.53
**Socioeconomic status**[1]									
High	402	3.48	12.44	6.97	3.48	4.23	7.96	0.75	14.68
Moderate	11,661	4.26	21.46	12.07	9.28	7.53	13.08	2.36	19.10
Low	29,758	6.86	30.12	20.12	16.80	13.46	21.33	5.32	29.07
Very low	3,305	6.66	32.86	23.48	16.13	19.18	22.21	5.75	37.40

[1]Stratified into four categories of pharmaceutical copayment: Very low (unemployed or recipient of social rescue aids), low (annual income < 18,000 €), moderate (annual income 18,000 to 100,000 €), and high (annual income >100,000 €).

Categorical outcomes were transformed to binary variables using the 95th percentile of the given variable among the target population as cutoff: hospital admission (i.e., one or more admissions), non-scheduled hospitalization (i.e., one or more admissions), admission to a skilled nursing facility for intermediate care (i.e., one or more admissions), admission to emergency room (i.e., more than two admissions), visit to primary care services (i.e., more than 21 visits), medication use (i.e., dispensation of more than 13 drugs belonging to different 5-digit group of the anatomic-therapeutic classification), expenditure (.i.e., healthcare cost above 4,315.1 €)

Table S4. Characteristics of the study population and rate of occurrence of each of the investigated outcomes. Subgroup **dementia**. Outcome results are presented as percentages.

	No.	All-cause death	Hospitalization	Non-scheduled hospitalization	Primary care visits	ER utilization	Medication use	Admission to skilled nursing facility	Expenditure
**Gender**									
Male	33,278	19.40	36.16	30.22	28.85	17.06	29.97	17.85	35.05
Female	67,508	16.22	27.34	23.19	24.52	11.83	27.62	14.43	24.97
**Age group**									
0-14	123	0.00	11.38	8.13	6.50	11.38	1.63	0.00	7.32
15-24	155	1.29	9.68	5.16	2.58	13.55	1.94	0.00	14.19
25-34	261	0.38	10.34	6.51	6.90	13.79	3.83	0.38	16.86
35-39	238	2.10	12.18	7.56	13.45	15.97	7.98	1.68	22.69
40-44	376	0.80	15.16	6.91	12.77	9.57	8.24	2.66	22.87
45-49	519	2.89	18.69	12.14	13.29	14.64	9.44	5.01	26.97
50-54	665	2.71	19.40	12.18	16.99	15.34	14.44	6.62	28.27
55-59	1,018	4.03	21.51	15.42	16.11	12.08	15.91	10.22	28.49
60-64	1,738	6.33	21.29	15.02	15.48	10.82	17.95	8.57	26.99
65-69	3,379	6.78	26.96	19.09	19.24	13.82	24.39	11.19	29.48
70-74	6,964	7.73	27.61	19.60	23.48	13.48	28.33	10.74	29.54
75-79	11,795	10.76	30.74	22.90	26.46	14.72	31.25	13.29	30.65
80-84	23,749	14.11	30.92	25.34	28.60	14.19	31.75	15.23	30.10
85-89	27,575	19.25	32.03	28.49	28.01	13.77	30.39	17.14	28.67
90-94	16,809	27.24	31.89	29.88	26.09	12.95	26.37	19.63	26.19
>95	5,422	35.76	28.37	26.93	20.73	9.98	20.23	18.57	20.20
**Socioeconomic status**[1]									
High	313	10.86	16.61	14.06	14.06	6.71	14.70	5.43	14.70
Moderate	17,054	15.19	27.72	22.84	23.43	11.63	24.96	12.27	25.84
Low	79,902	17.82	30.81	26.08	26.59	13.82	29.13	16.24	28.70
Very low	3,517	15.58	31.02	26.39	24.71	17.40	29.54	16.83	32.27

[1]Stratified into four categories of pharmaceutical copayment: Very low (unemployed or recipient of social rescue aids), low (annual income < 18,000 €), moderate (annual income 18,000 to 100,000 €), and high (annual income >100,000 €).

Categorical outcomes were transformed to binary variables using the 95th percentile of the given variable among the target population as cutoff: hospital admission (i.e., one or more admissions), non-scheduled hospitalization (i.e., one or more admissions), admission to a skilled nursing facility for intermediate care (i.e., one or more admissions), admission to emergency room (i.e., more than two admissions), visit to primary care services (i.e., more than 21 visits), medication use (i.e., dispensation of more than 13 drugs belonging to different 5-digit group of the anatomic-therapeutic classification), expenditure (.i.e., healthcare cost above 4,315.1 €)

Table S5. Characteristics of the study population and rate of occurrence of each of the investigated outcomes. Subgroup **diabetes**. Outcome results are presented as percentages.

	No.	All-cause death	Hospitalization	Non-scheduled hospitalization	Primary care visits	ER utilization	Medication use	Admission to skilled nursing facility	Expenditure
**Gender**									
Male	318,799	3.85	21.60	12.75	17.36	8.03	19.95	3.12	19.28
Female	269,722	4.09	21.44	12.26	22.56	8.75	28.61	4.28	18.81
**Age group**									
0-14	2,518	0.08	16.28	13.22	16.00	11.60	0.52	0.00	10.64
15-24	3,455	0.17	9.99	7.50	6.60	9.18	1.68	0.03	9.00
25-34	6,447	0.08	12.95	8.22	7.71	10.24	3.52	0.05	9.97
35-39	8,065	0.17	11.52	6.72	7.74	7.85	5.34	0.17	9.99
40-44	14,401	0.33	9.96	5.29	8.83	6.73	7.27	0.22	9.82
45-49	21,523	0.49	10.63	5.23	9.66	5.97	8.96	0.35	10.65
50-54	34,583	0.74	12.21	6.05	10.70	6.06	11.45	0.61	12.91
55-59	49,858	0.96	14.60	7.02	11.60	5.76	13.72	0.83	13.94
60-64	64,213	1.31	16.42	7.58	12.70	5.75	16.89	1.14	15.15
65-69	78,720	1.79	19.11	9.01	14.50	6.58	21.49	1.54	17.08
70-74	86,300	2.34	22.49	10.92	18.90	7.67	26.87	2.39	19.76
75-79	71,275	3.87	27.06	14.61	25.22	9.63	32.44	4.21	23.30
80-84	72,023	6.64	29.57	19.21	31.10	11.42	35.94	7.00	25.76
85-89	49,702	11.02	31.23	24.20	34.29	12.99	36.71	10.43	26.93
90-94	20,559	18.24	31.12	27.18	33.20	12.67	32.68	13.55	25.34
>95	4,879	27.26	29.74	26.97	30.31	9.59	27.73	15.23	21.93
**Socioeconomic status**[1]									
High	3,188	2.57	9.57	5.74	5.14	2.54	11.14	0.69	9.41
Moderate	152,299	2.64	17.73	9.22	13.55	5.75	17.62	2.00	14.83
Low	404,692	4.51	23.08	13.79	22.02	9.12	25.99	4.32	20.51
Very low	28,342	3.21	21.13	12.93	22.16	12.22	29.71	3.39	22.21

[1]Stratified into four categories of pharmaceutical copayment: Very low (unemployed or recipient of social rescue aids), low (annual income < 18,000 €), moderate (annual income 18,000 to 100,000 €), and high (annual income >100,000 €).

Categorical outcomes were transformed to binary variables using the 95th percentile of the given variable among the target population as cutoff: hospital admission (i.e., one or more admissions), non-scheduled hospitalization (i.e., one or more admissions), admission to a skilled nursing facility for intermediate care (i.e., one or more admissions), admission to emergency room (i.e., more than two admissions), visit to primary care services (i.e., more than 21 visits), medication use (i.e., dispensation of more than 13 drugs belonging to different 5-digit group of the anatomic-therapeutic classification), expenditure (.i.e., healthcare cost above 4,315.1 €)

Table S6. Characteristics of the study population and rate of occurrence of each of the investigated outcomes. Subgroup **heart failure**. Outcome results are presented as percentages.

	No.	All-cause death	Hospitalization	Non-scheduled hospitalization	Primary care visits	ER utilization	Medication use	Admission to skilled nursing facility	Expenditure
**Gender**									
Male	89,974	12.96	44.36	35.05	37.13	19.96	41.22	10.01	42.81
Female	120,723	11.34	35.88	28.57	36.05	16.50	39.88	10.92	32.31
**Age group**									
0-14	1,564	1.02	21.74	12.53	10.49	16.05	1.34	0.00	18.54
15-24	1,336	0.75	10.78	6.81	2.92	11.00	3.22	0.07	8.23
25-34	4,308	0.21	11.84	8.43	4.27	9.33	2.21	0.14	5.92
35-39	2,645	0.60	16.14	10.36	7.18	10.40	5.37	0.34	12.36
40-44	3,290	0.88	15.93	9.33	8.24	9.09	8.72	0.58	14.22
45-49	3,941	1.37	20.76	14.49	12.21	11.27	13.25	0.91	21.14
50-54	5,476	2.79	25.35	17.62	15.74	12.91	19.43	1.64	26.44
55-59	7,584	3.48	30.67	21.65	19.87	13.05	25.99	2.51	32.27
60-64	10,236	4.77	34.43	23.62	24.36	14.69	32.53	3.58	35.32
65-69	15,131	5.88	37.49	26.23	29.20	16.71	39.30	4.75	38.21
70-74	21,355	6.49	40.41	28.61	35.63	17.68	46.23	6.03	40.86
75-79	24,718	9.03	44.32	32.72	42.35	20.43	50.96	9.09	43.30
80-84	38,854	12.21	44.84	35.65	46.01	20.91	50.17	12.39	42.29
85-89	39,105	17.38	44.68	38.70	45.99	20.59	46.87	16.41	40.23
90-94	23,404	24.65	43.02	39.41	41.47	18.14	39.01	19.17	35.11
>95	7,750	32.23	38.84	36.45	34.45	14.04	30.71	19.28	27.72
**Socioeconomic status**[1]									
High	682	10.12	26.10	19.50	18.77	9.53	27.42	3.08	26.10
Moderate	40,646	9.66	35.58	26.77	29.60	14.54	33.99	7.07	32.56
Low	160,801	12.75	40.53	32.51	38.45	18.63	41.92	11.49	37.70
Very low	8,568	9.92	39.93	31.97	34.31	22.76	44.54	9.52	40.77

[1]Stratified into four categories of pharmaceutical copayment: Very low (unemployed or recipient of social rescue aids), low (annual income < 18,000 €), moderate (annual income 18,000 to 100,000 €), and high (annual income >100,000 €).

Categorical outcomes were transformed to binary variables using the 95th percentile of the given variable among the target population as cutoff: hospital admission (i.e., one or more admissions), non-scheduled hospitalization (i.e., one or more admissions), admission to a skilled nursing facility for intermediate care (i.e., one or more admissions), admission to emergency room (i.e., more than two admissions), visit to primary care services (i.e., more than 21 visits), medication use (i.e., dispensation of more than 13 drugs belonging to different 5-digit group of the anatomic-therapeutic classification), expenditure (.i.e., healthcare cost above 4,315.1 €)

Table S7. Characteristics of the study population and rate of occurrence of each of the investigated outcomes. Subgroup **chronic kidney disease**. Outcome results are presented as percentages.

	No.	All-cause death	Hospitalization	Non-scheduled hospitalization	Primary care visits	ER utilization	Medication use	Admission to skilled nursing facility	Expenditure
**Gender**									
Male	132,274	8.62	33.59	23.25	24.86	13.58	29.39	6.68	31.77
Female	152,599	8.07	28.61	20.08	28.14	11.93	32.20	8.04	25.39
**Age group**									
0-14	659	0.61	28.68	20.03	11.53	20.49	6.68	0.00	28.38
15-24	739	0.68	26.52	17.59	3.92	15.83	6.36	0.14	25.17
25-34	1,622	0.68	21.95	13.13	5.98	13.75	8.38	0.18	26.57
35-39	1,818	0.39	22.17	13.53	6.82	11.99	11.11	0.66	30.36
40-44	2,870	1.08	21.39	12.65	7.94	11.08	13.34	0.59	29.76
45-49	3,960	1.54	22.88	13.06	10.30	10.66	15.71	1.09	30.20
50-54	6,035	2.17	24.52	14.32	11.07	10.65	18.87	1.44	32.43
55-59	8,803	2.64	25.72	15.39	13.17	11.68	22.13	2.00	31.10
60-64	13,491	3.11	25.85	14.62	14.62	10.36	24.78	2.25	28.46
65-69	22,042	3.48	27.86	16.10	17.00	11.16	27.98	2.87	27.71
70-74	33,782	3.77	28.99	16.70	21.04	11.12	30.92	3.60	27.32
75-79	39,223	5.33	31.97	19.64	26.71	12.81	34.52	5.53	29.22
80-84	57,212	7.74	32.98	22.76	31.82	13.70	35.63	8.09	29.12
85-89	54,356	11.74	33.46	26.46	34.54	14.04	34.31	11.18	28.61
90-94	29,255	18.65	33.58	29.62	34.05	13.48	29.92	14.77	26.74
>95	9,006	26.89	31.50	29.35	30.93	11.35	24.53	15.87	22.15
**Socioeconomic status**[1]									
High	1,042	4.61	18.81	12.28	8.73	5.66	16.60	1.73	21.31
Moderate	61,758	6.05	26.92	17.16	19.41	9.77	24.56	4.54	24.25
Low	211,820	9.05	32.03	22.76	28.77	13.32	32.51	8.27	29.21
Very low	10,253	7.47	33.28	23.95	27.23	18.19	37.24	7.59	36.08

[1]Stratified into four categories of pharmaceutical copayment: Very low (unemployed or recipient of social rescue aids), low (annual income < 18,000 €), moderate (annual income 18,000 to 100,000 €), and high (annual income >100,000 €).

Categorical outcomes were transformed to binary variables using the 95th percentile of the given variable among the target population as cutoff: hospital admission (i.e., one or more admissions), non-scheduled hospitalization (i.e., one or more admissions), admission to a skilled nursing facility for intermediate care (i.e., one or more admissions), admission to emergency room (i.e., more than two admissions), visit to primary care services (i.e., more than 21 visits), medication use (i.e., dispensation of more than 13 drugs belonging to different 5-digit group of the anatomic-therapeutic classification), expenditure (.i.e., healthcare cost above 4,315.1 €)

Table S8. Characteristics of the study population and rate of occurrence of each of the investigated outcomes. Subgroup **chronic obstructive pulmonary disease**. Outcome results are presented as percentages.

	No.	All-cause death	Hospitalization	Non-scheduled hospitalization	Primary care visits	ER utilization	Medication use	Admission to skilled nursing facility	Expenditure
**Gender**									
Male	216,963	5.93	28.06	18.52	20.16	12.08	24.80	4.69	25.05
Female	141,026	4.94	25.08	16.45	22.85	12.66	30.72	5.36	22.43
**Age group**									
0-14	13,767	0.03	7.11	4.12	7.02	14.47	0.52	0.00	2.96
15-24	5,047	0.20	7.03	4.28	2.56	9.71	1.62	0.08	5.03
25-34	5,934	0.15	12.30	7.89	5.34	12.62	3.35	0.10	7.40
35-39	5,743	0.30	12.50	7.54	6.95	11.20	5.69	0.16	9.09
40-44	8,868	0.64	12.37	6.71	8.15	9.10	7.07	0.30	10.98
45-49	12,346	0.88	14.23	7.39	9.27	9.25	10.36	0.50	14.39
50-54	18,854	1.38	17.04	9.35	10.76	8.92	13.19	0.90	17.11
55-59	26,953	1.77	19.62	11.04	11.46	8.33	15.80	1.34	18.62
60-64	33,670	2.30	21.62	12.09	13.36	8.63	19.52	1.77	19.62
65-69	41,781	2.97	24.92	13.92	15.71	9.51	25.09	2.37	22.04
70-74	47,067	3.69	28.87	16.67	20.43	11.28	31.15	3.34	25.73
75-79	40,738	5.64	34.14	21.25	27.92	13.94	38.28	5.53	30.80
80-84	45,631	8.82	36.86	26.29	34.60	16.25	41.79	8.87	33.50
85-89	32,745	13.63	38.96	31.97	38.15	17.90	43.32	13.43	35.35
90-94	14,916	21.37	39.68	35.36	37.25	17.50	39.90	17.00	33.15
>95	3,929	29.75	36.50	33.67	32.58	14.53	32.88	17.97	27.74
**Socioeconomic status**[1]									
High	1,668	3.72	12.23	7.73	5.64	4.08	12.05	0.84	11.45
Moderate	91,281	3.72	21.71	12.86	14.75	8.50	19.95	2.74	18.11
Low	246,130	6.30	28.89	19.51	23.74	13.41	29.70	5.83	25.96
Very low	18,910	4.60	27.12	18.49	21.02	17.13	29.77	4.60	28.36

[1]Stratified into four categories of pharmaceutical copayment: Very low (unemployed or recipient of social rescue aids), low (annual income < 18,000 €), moderate (annual income 18,000 to 100,000 €), and high (annual income >100,000 €).

Categorical outcomes were transformed to binary variables using the 95th percentile of the given variable among the target population as cutoff: hospital admission (i.e., one or more admissions), non-scheduled hospitalization (i.e., one or more admissions), admission to a skilled nursing facility for intermediate care (i.e., one or more admissions), admission to emergency room (i.e., more than two admissions), visit to primary care services (i.e., more than 21 visits), medication use (i.e., dispensation of more than 13 drugs belonging to different 5-digit group of the anatomic-therapeutic classification), expenditure (.i.e., healthcare cost above 4,315.1 €)

Table S9. Characteristics of the study population and rate of occurrence of each of the investigated outcomes. Subgroup **older than 64 years institutionalized in a nursing home for long-term care**. Outcome results are presented as percentages.

	No.	All-cause death	Hospitalization	Non-scheduled hospitalization	Primary care visits	ER utilization	Medication use	Admission to skilled nursing facility	Expenditure
**Gender**									
Male	18,814	23.68	37.37	32.81	19.91	16.19	36.52	15.68	32.58
Female	48,642	19.21	27.84	24.87	15.87	10.42	33.13	12.04	22.45
**Age group**									
65-69	2,632	8.40	26.63	20.10	15.43	12.88	29.33	9.88	27.85
70-74	3,964	10.22	27.22	21.54	17.33	12.46	33.60	10.82	28.30
75-79	5,590	14.67	33.02	27.42	19.36	14.47	37.48	14.78	31.09
80-84	12,587	17.25	32.52	28.16	19.39	13.85	38.52	14.09	29.40
85-89	18,934	20.53	32.41	29.34	18.33	12.52	36.55	13.90	26.52
90-94	16,383	24.73	29.69	27.54	15.67	10.90	31.81	12.61	21.69
>95	7,366	30.45	25.14	23.59	11.04	7.78	24.50	11.15	16.02
**Socioeconomic status[1]**									
High	124	16.13	24.19	21.77	14.52	6.45	33.06	8.87	20.97
Moderate	8,606	19.61	29.08	25.28	15.91	11.82	33.89	12.03	25.02
Low	55,944	20.77	30.80	27.46	17.32	12.07	34.20	13.26	25.31
Very low	2,782	16.89	29.12	25.27	14.09	12.15	32.06	12.37	25.63

[1]Stratified into four categories of pharmaceutical copayment: Very low (unemployed or recipient of social rescue aids), low (annual income < 18,000 €), moderate (annual income 18,000 to 100,000 €), and high (annual income >100,000 €).

Categorical outcomes were transformed to binary variables using the 95th percentile of the given variable among the target population as cutoff: hospital admission (i.e., one or more admissions), non-scheduled hospitalization (i.e., one or more admissions), admission to a skilled nursing facility for intermediate care (i.e., one or more admissions), admission to emergency room (i.e., more than two admissions), visit to primary care services (i.e., more than 21 visits), medication use (i.e., dispensation of more than 13 drugs belonging to different 5-digit group of the anatomic-therapeutic classification), expenditure (.i.e., healthcare cost above 4,315.1 €)

PERFORMANCE OF MULTIMORBIDITY MEASUREMENTS IN ALL POPULATIONS

Tables S10 to S19 show the result of the performance analysis of each model for all statistical indicators and investigated outcomes. Values correspond to the result of each statistic: Akaike information criteria (AIC), pR^2, area under the receiving operating characteristics courve (AUC ROC), area under the precision recall courve (AUC_PR). In all tables, cells are colored according to the goodness of fit of each model for a given statistic, from red (worse) to strong green (best). Tables S10 to S19 represent the following populations, respectively: general population adults (>17 years), general population aged >65 years, ischemic heart disease, cirrhosis, dementia, diabetes, heart failure, chronic kidney disease, chronic obstructive pulmonary disease, older than 64 years institutionalized in a nursing home for long-term care.

Table S10: Performance of each model for multimorbidity measurement for explaining the selected outcomes, measured using four statistics. General adult population (i.e., aged ≥ 18 years)

		**Age + Gender + socioeconomic status**	**+ CCI**	**+ QOF**	**+ Karolinska**	**+ HCUP**	**+ GMA**
**All-cause death**	AIC	54.8	47.1	50.4	51.3	49.9	45.5
	pR^2	25.2	35.8	31.2	30.1	31.9	37.9
	AUC ROC	0.902	0.947	0.928	0.925	0.933	0.952
	AUC_PR	0.097	0.202	0.164	0.149	0.165	0.237
**Hospitalization**	AIC	347.2	328.1	329.2	315.9	314.2	282.9
	pR^2	7.5	12.6	12.3	15.9	16.3	24.6
	AUC ROC	0.700	0.751	0.749	0.788	0.789	0.846
	AUC_PR	0.194	0.287	0.281	0.318	0.328	0.460
**Non-scheduled hospitalization**	AIC	205.1	188.2	187.8	183.8	181.1	151.3
	pR^2	9.5	17.0	17.2	19.0	20.1	33.3
	AUC ROC	0.736	0.801	0.800	0.818	0.825	0.897
	AUC_PR	0.132	0.236	0.243	0.259	0.275	0.464
**Skilled Nursing facility admision**	AIC	47.5	42.2	42.8	41.9	40.8	35.8
	pR^2	25.5	33.9	32.9	34.3	36.1	43.9
	AUC ROC	0.912	0.948	0.942	0.947	0.953	0.971
	AUC_PR	0.078	0.154	0.155	0.167	0.184	0.291
**ER utilization (>2 visits)**	AIC	208.7	200.3	198.4	188.4	188.4	173.9
	pR^2	3.7	7.6	8.5	13.0	13.1	19.8
	AUC ROC	0.655	0.714	0.723	0.781	0.780	0.838
	AUC_PR	0.077	0.123	0.133	0.174	0.178	0.275
**PC utilization (> 21 visits)**	AIC	214.1	203.4	193.9	182.8	184.6	179.1
	pR^2	16.4	20.6	24.3	28.7	27.9	30.1
	AUC ROC	0.803	0.836	0.857	0.890	0.887	0.904
	AUC_PR	0.197	0.258	0.314	0.354	0.344	0.386
**Medications (>13 drugs)**	AIC	203.2	180.1	167.1	149.7	150.1	148.2
	pR^2	21.0	30.0	35.1	41.8	41.7	42.4
	AUC ROC	0.845	0.896	0.915	0.939	0.939	0.945
	AUC_PR	0.203	0.350	0.428	0.506	0.504	0.530
**Expenditure (>4,315.1 €)**	AIC	249.9	214.1	221.6	208.5	204.0	176.4
	pR^2	10.6	23.4	20.7	25.4	27.0	36.9
	AUC ROC	0.750	0.842	0.828	0.862	0.870	0.912
	AUC_PR	0.159	0.356	0.313	0.365	0.388	0.541

AUC_PR: area under the precision-recall. **AIC:** Akaike information criterion. **CCI:** Charlson Comorbidity Index. **ER:** emergency room. **GMA:** adjusted morbidity groups. **HCUP:** healthcare cost and utilization project of the US Agency for Healthcare Research and Quality. **PC:** primary care (>21 visits including general practitioner, nurse, and social worker, either at the primary care facility, home or via teleconsultation. **pR^2**: pseudo-R squared.
Cells are colored according to the goodness of fit of each model for a given statistic, from red (worse) to strong green (best).

Table S11: Performance of each model for multimorbidity measurement for explaining the selected outcomes, measured using four statistics. People aged >64 years

	Statistic	Age + Gender + socioeconomic status	+ CCI	+ QOF	+ Karolinska	+ HCUP	+ GMA
**All-cause death**	AIC	42.6	37.3	39.2	40.0	39.0	35.8
	pR^2	12.6	23.5	19.5	18.0	20.0	26.5
	AUC ROC	0.781	0.871	0.842	0.830	0.845	0.887
	AUC_PR	0.137	0.232	0.198	0.184	0.201	0.270
**Hospitalization**	AIC	140.3	130.3	130.3	125.0	124.0	108.4
	pR^2	2.4	9.3	9.3	13.0	13.7	24.6
	AUC ROC	0.609	0.711	0.709	0.749	0.755	0.823
	AUC_PR	0.255	0.386	0.384	0.425	0.438	0.593
**Non-scheduled hospitalization**	AIC	92.2	81.7	80.6	79.0	77.4	59.8
	pR^2	5.7	16.4	17.6	19.3	20.9	38.8
	AUC ROC	0.681	0.797	0.800	0.812	0.823	0.908
	AUC_PR	0.185	0.330	0.349	0.367	0.388	0.620
**Skilled Nursing facility admision**	AIC	39.9	36.0	36.1	35.5	34.6	30.5
	pR^2	9.6	18.4	18.2	19.7	21.7	31.0
	AUC ROC	0.756	0.843	0.837	0.848	0.863	0.913
	AUC_PR	0.090	0.166	0.169	0.182	0.199	0.309
**ER utilization (>2 visits)**	AIC	70.1	65.1	64.2	60.8	60.7	53.8
	pR^2	2.4	9.3	10.6	15.3	15.5	25.1
	AUC ROC	0.622	0.737	0.744	0.794	0.795	0.859
	AUC_PR	0.098	0.180	0.199	0.246	0.251	0.386
**PC utilization (> 21 visits)**	AIC	117.9	111.5	105.8	101.5	102.8	99.1
	pR^2	5.9	11.1	15.6	19.0	18.0	20.9
	AUC ROC	0.676	0.745	0.784	0.810	0.804	0.833
	AUC_PR	0.246	0.322	0.386	0.422	0.410	0.455
**Medications (>13 drugs)**	AIC	128.8	114.7	106.8	97.5	98.0	96.3
	pR^2	3.5	14.1	20.0	27.0	26.6	27.8
	AUC ROC	0.633	0.776	0.811	0.853	0.852	0.867
	AUC_PR	0.234	0.401	0.479	0.552	0.549	0.574

**Expenditure (>4,315.1 €)**	AIC	118.0	102.1	102.9	97.3	95.5	79.7
	pR^2	2.7	15.8	15.2	19.8	21.2	34.3
	AUC ROC	0.620	0.782	0.772	0.806	0.817	0.881
	AUC_PR	0.202	0.407	0.395	0.450	0.469	0.636

AUC_PR: area under the precision-recall. **AIC:** Akaike information criterion. **CCI:** Charlson Comorbidity Index. **ER:** emergency room. **GMA:** adjusted morbidity groups. **HCUP:** healthcare cost and utilization project of the US Agency for Healthcare Research and Quality. **PC:** primary care (>21 visits including general practitioner, nurse, and social worker, either at the primary care facility, home or via teleconsultation. **pR^2**: pseudo-R squared.

Cells are colored according to the goodness of fit of each model for a given statistic, from red (worse) to strong green (best).

Table S12: Performance of each model for multimorbidity measurement for explaining the selected outcomes, measured using four statistics. Ischemic heart disease

	Statistic	Age + Gender + socioeconomic status	+ CCI	+ QOF	+ Karolinska	+ HCUP	+ GMA
**All-cause death**	AIC	10.3	9.2	9.6	9.7	9.5	8.8
	pR^2	11.1	20.5	17.7	16.1	18.2	24.1
	AUC ROC	0.756	0.840	0.820	0.806	0.823	0.864
	AUC_PR	0.174	0.265	0.235	0.222	0.240	0.301
**Hospitalization**	AIC	29.7	27.7	27.8	26.9	26.6	22.8
	pR^2	1.7	8.1	7.9	10.8	11.8	24.3
	AUC ROC	0.588	0.692	0.687	0.719	0.729	0.815
	AUC_PR	0.368	0.501	0.499	0.536	0.550	0.699
**Non-scheduled hospitalization**	AIC	24.6	22.6	22.5	22.1	21.7	17.7
	pR^2	2.7	10.5	11.0	12.5	14.1	30.0
	AUC ROC	0.617	0.726	0.726	0.740	0.754	0.852
	AUC_PR	0.286	0.420	0.432	0.454	0.473	0.664
**Skilled Nursing facility admision**	AIC	9.0	8.2	8.1	8.0	7.8	6.9
	pR^2	10.8	18.9	19.2	20.4	22.7	31.5
	AUC ROC	0.759	0.837	0.838	0.844	0.860	0.906
	AUC_PR	0.128	0.204	0.213	0.230	0.250	0.359
**ER utilization (>2 visits)**	AIC	18.2	17.2	16.9	16.1	16.1	14.3
	pR^2	2.0	7.8	9.1	13.5	13.6	23.4
	AUC ROC	0.603	0.708	0.718	0.763	0.764	0.832
	AUC_PR	0.167	0.259	0.281	0.336	0.339	0.477
**PC utilization (> 21 visits)**	AIC	25.2	24.2	23.2	22.5	22.7	22.1
	pR^2	6.2	10.0	13.7	16.4	15.5	18.0
	AUC ROC	0.671	0.722	0.758	0.780	0.772	0.796
	AUC_PR	0.351	0.412	0.468	0.499	0.488	0.525
**Medications (>13 drugs)**	AIC	29.0	26.4	25.4	23.6	23.8	23.3
	pR^2	6.3	14.8	17.8	23.6	23.2	24.6
	AUC ROC	0.660	0.762	0.781	0.820	0.818	0.830
	AUC_PR	0.448	0.578	0.617	0.674	0.670	0.689

**Expenditure (>4,315.1 €)**	AIC	28.6	25.5	25.7	24.5	24.0	20.2
	pR^2	1.6	12.5	11.6	16.0	17.5	30.6
	AUC ROC	0.584	0.739	0.728	0.763	0.775	0.851
	AUC_PR	0.337	0.535	0.524	0.579	0.596	0.731

AUC_PR: area under the precision-recall. **AIC:** Akaike information criterion. **CCI:** Charlson Comorbidity Index. **ER:** emergency room. **GMA:** adjusted morbidity groups. **HCUP:** healthcare cost and utilization project of the US Agency for Healthcare Research and Quality. **PC:** primary care (>21 visits including general practitioner, nurse, and social worker, either at the primary care facility, home or via teleconsultation. **pR^2**: pseudo-R squared.

Cells are colored according to the goodness of fit of each model for a given statistic, from red (worse) to strong green (best).

Table S13: Performance of each model for multimorbidity measurement for explaining the selected outcomes, measured using four statistics. Cirrhosis

	Statistic	Age + Gender + socioeconomic status	+ CCI	+ QOF	+ Karolinska	+ HCUP	+ GMA
**All-cause death**	AIC	1.9	1.6	1.8	1.8	1.8	1.6
	pR^2	9.2	23.4	14.6	14.4	16.4	26.1
	AUC ROC	0.731	0.862	0.790	0.790	0.809	0.884
	AUC_PR	0.157	0.297	0.214	0.207	0.227	0.323
**Hospitalization**	AIC	5.1	4.5	4.8	4.5	4.5	3.7
	pR^2	4.8	15.4	10.9	15.4	16.2	31.2
	AUC ROC	0.648	0.768	0.722	0.764	0.770	0.857
	AUC_PR	0.390	0.558	0.506	0.556	0.571	0.735
**Non-scheduled hospitalization**	AIC	4.1	3.5	3.7	3.5	3.5	2.6
	pR^2	5.6	18.0	13.7	17.6	19.0	38.5
	AUC ROC	0.666	0.802	0.754	0.790	0.798	0.901
	AUC_PR	0.290	0.459	0.428	0.466	0.488	0.706
**Skilled Nursing facility admision**	AIC	1.5	1.3	1.4	1.4	1.3	1.2
	pR^2	10.4	21.3	17.6	19.7	21.6	30.6
	AUC ROC	0.755	0.857	0.827	0.843	0.858	0.910
	AUC_PR	0.124	0.217	0.191	0.211	0.227	0.327
**ER utilization (>2 visits)**	AIC	3.3	3.0	3.1	2.9	2.9	2.5
	pR^2	3.3	10.7	9.6	14.9	15.3	26.4
	AUC ROC	0.632	0.745	0.723	0.778	0.780	0.855
	AUC_PR	0.183	0.284	0.284	0.343	0.355	0.505
**PC utilization (> 21 visits)**	AIC	3.5	3.3	3.2	3.1	3.1	3.0
	pR^2	7.7	12.1	15.2	18.9	18.3	20.8
	AUC ROC	0.699	0.752	0.778	0.808	0.805	0.828
	AUC_PR	0.268	0.327	0.380	0.415	0.409	0.447
**Medications (>13 drugs)**	AIC	4.0	3.6	3.4	3.1	3.1	3.1
	pR^2	9.0	18.0	23.5	29.4	29.6	30.2
	AUC ROC	0.706	0.797	0.831	0.864	0.866	0.875
	AUC_PR	0.326	0.455	0.540	0.595	0.596	0.609

**Expenditure (>4,315.1 €)**	AIC	5.0	4.1	4.5	4.2	4.1	3.3
	pR^2	5.1	23.3	14.8	20.3	22.6	37.3
	AUC ROC	0.649	0.825	0.759	0.799	0.814	0.887
	AUC_PR	0.373	0.635	0.543	0.602	0.630	0.772

AUC_PR: area under the precision-recall. **AIC:** Akaike information criterion. **CCI:** Charlson Comorbidity Index. **ER:** emergency room. **GMA:** adjusted morbidity groups. **HCUP:** healthcare cost and utilization project of the US Agency for Healthcare Research and Quality. **PC:** primary care (>21 visits including general practitioner, nurse, and social worker, either at the primary care facility, home or via teleconsultation. **pR^2**: pseudo-R squared.

Cells are colored according to the goodness of fit of each model for a given statistic, from red (worse) to strong green (best).

Table S14: Performance of each model for multimorbidity measurement for explaining the selected outcomes, measured using four statistics. Dementia

	Statistic	Age + Gender + socioeconomic status	+ CCI	+ QOF	+ Karolinska	+ HCUP	+ GMA
**All-cause death**	AIC	8.8	8.5	8.6	8.6	8.6	8.2
	pR^2	5.8	8.7	7.8	7.4	8.0	12.0
	AUC ROC	0.668	0.708	0.698	0.693	0.700	0.747
	AUC_PR	0.281	0.325	0.310	0.305	0.311	0.364
**Hospitalization**	AIC	12.2	11.4	11.4	11.1	11.0	8.8
	pR^2	1.5	7.5	7.7	10.7	11.1	29.3
	AUC ROC	0.577	0.687	0.687	0.720	0.725	0.847
	AUC_PR	0.362	0.484	0.487	0.522	0.531	0.729
**Non-scheduled hospitalization**	AIC	11.2	10.5	10.4	10.2	10.1	7.7
	pR^2	2.0	8.4	9.2	11.4	12.2	32.9
	AUC ROC	0.593	0.703	0.706	0.729	0.738	0.870
	AUC_PR	0.320	0.438	0.451	0.476	0.489	0.715
**Skilled Nursing facility admision**	AIC	8.6	8.2	8.2	8.1	8.0	7.3
	pR^2	2.0	5.7	5.6	7.3	8.2	16.2
	AUC ROC	0.595	0.673	0.670	0.695	0.707	0.789
	AUC_PR	0.202	0.266	0.264	0.286	0.298	0.403
**ER utilization (>2 visits)**	AIC	7.9	7.5	7.4	7.1	7.1	6.1
	pR^2	1.1	6.2	7.6	11.3	11.4	23.8
	AUC ROC	0.575	0.687	0.699	0.741	0.742	0.835
	AUC_PR	0.170	0.253	0.276	0.319	0.323	0.488
**PC utilization (> 21 visits)**	AIC	11.4	11.1	10.8	10.5	10.6	10.2
	pR^2	1.2	4.4	6.3	9.1	8.3	11.9
	AUC ROC	0.567	0.652	0.675	0.710	0.701	0.746
	AUC_PR	0.303	0.375	0.409	0.443	0.433	0.483

**Medications (>13 drugs)**	AIC	11.9	11.0	10.4	9.9	9.9	9.7
	pR^2	1.4	9.0	13.4	17.7	17.6	19.1
	AUC ROC	0.568	0.714	0.747	0.781	0.781	0.796
	AUC_PR	0.325	0.477	0.536	0.583	0.582	0.605
**Expenditure (>4,315.1 €)**	AIC	11.8	11.0	10.9	10.4	10.3	8.5
	pR^2	1.5	8.7	9.3	13.6	14.1	29.8
	AUC ROC	0.580	0.702	0.705	0.747	0.751	0.851
	AUC_PR	0.344	0.484	0.489	0.542	0.549	0.718

AUC_PR: area under the precision-recall. **AIC:** Akaike information criterion. **CCI:** Charlson Comorbidity Index. **ER:** emergency room. **GMA:** adjusted morbidity groups. **HCUP:** healthcare cost and utilization project of the US Agency for Healthcare Research and Quality. **PC:** primary care (>21 visits including general practitioner, nurse, and social worker, either at the primary care facility, home or via teleconsultation. **pR^2**: pseudo-R squared.

Cells are colored according to the goodness of fit of each model for a given statistic, from red (worse) to strong green (best).

Table S15: Performance of each model for multimorbidity measurement for explaining the selected outcomes, measured using four statistics. Diabetes

	Statistic	Age + Gender + socioeconomic status	+ CCI	+ QOF	+ Karolinska	+ HCUP	+ GMA
**All-cause death**	AIC	17.0	14.7	15.5	15.8	15.3	14.0
	pR^2	13.3	25.2	20.8	19.7	22.2	28.7
	AUC ROC	0.788	0.882	0.852	0.844	0.862	0.901
	AUC_PR	0.142	0.246	0.207	0.196	0.217	0.283
**Hospitalization**	AIC	59.3	54.7	55.1	52.9	52.1	45.3
	pR^2	3.2	10.8	10.2	13.8	15.0	26.1
	AUC ROC	0.626	0.728	0.717	0.754	0.764	0.831
	AUC_PR	0.296	0.436	0.429	0.470	0.489	0.637
**Non-scheduled hospitalization**	AIC	42.0	37.2	36.9	36.1	35.1	27.5
	pR^2	5.3	16.2	16.8	18.6	21.0	38.2
	AUC ROC	0.671	0.790	0.789	0.802	0.819	0.903
	AUC_PR	0.216	0.367	0.385	0.404	0.432	0.645
**Skilled Nursing facility admision**	AIC	16.3	14.6	14.8	14.5	14.0	12.4
	pR^2	11.7	20.8	20.1	21.7	24.3	32.9
	AUC ROC	0.777	0.860	0.851	0.861	0.878	0.921
	AUC_PR	0.105	0.184	0.187	0.202	0.224	0.327
**ER utilization (>2 visits)**	AIC	33.1	30.7	30.4	28.8	28.6	25.4
	pR^2	2.3	9.3	10.3	15.0	15.7	25.0
	AUC ROC	0.616	0.735	0.738	0.785	0.790	0.851
	AUC_PR	0.121	0.212	0.231	0.283	0.292	0.425
**PC utilization (> 21 visits)**	AIC	55.2	52.8	51.4	49.2	49.5	48.0
	pR^2	5.7	9.7	12.2	16.0	15.4	17.9
	AUC ROC	0.668	0.723	0.745	0.780	0.776	0.804
	AUC_PR	0.305	0.370	0.417	0.457	0.448	0.488
**Medications (>13 drugs)**	AIC	60.4	54.7	52.0	47.2	47.4	46.6
	pR^2	6.7	15.6	19.7	27.1	26.8	28.0
	AUC ROC	0.672	0.776	0.802	0.846	0.846	0.858

	AUC_PR	0.351	0.498	0.558	0.629	0.627	0.648
**Expenditure (>4,315.1 €)**	AIC	55.8	47.9	48.8	45.8	44.5	37.6
	pR^2	2.7	16.5	14.9	20.1	22.4	34.5
	AUC ROC	0.615	0.783	0.765	0.803	0.819	0.878
	AUC_PR	0.257	0.481	0.461	0.522	0.547	0.690

AUC_PR: area under the precision-recall. **AIC:** Akaike information criterion. **CCI:** Charlson Comorbidity Index. **ER:** emergency room. **GMA:** adjusted morbidity groups. **HCUP:** healthcare cost and utilization project of the US Agency for Healthcare Research and Quality. **PC:** primary care (>21 visits including general practitioner, nurse, and social worker, either at the primary care facility, home or via teleconsultation. **pR^2**: pseudo-R squared.

Cells are colored according to the goodness of fit of each model for a given statistic, from red (worse) to strong green (best).

Table S16: Performance of each model for multimorbidity measurement for explaining the selected outcomes, measured using four statistics. Heart failure

	Statistic	Age + Gender + socioeconomic status	+ CCI	+ QOF	+ Karolinska	+ HCUP	+ GMA
**All-cause death**	AIC	14.1	13.2	13.6	13.7	13.5	12.6
	pR^2	9.1	14.6	12.1	11.6	13.1	18.6
	AUC ROC	0.717	0.776	0.753	0.747	0.763	0.813
	AUC_PR	0.237	0.302	0.273	0.265	0.280	0.339
**Hospitalization**	AIC	27.4	26.1	26.2	25.3	25.0	20.3
	pR^2	3.2	7.8	7.5	10.4	11.7	28.2
	AUC ROC	0.603	0.685	0.681	0.713	0.725	0.837
	AUC_PR	0.470	0.569	0.564	0.605	0.619	0.785
**Non-scheduled hospitalization**	AIC	25.2	23.9	23.8	23.3	22.9	17.8
	pR^2	3.9	8.9	9.3	11.3	12.8	32.2
	AUC ROC	0.620	0.702	0.704	0.724	0.738	0.862
	AUC_PR	0.395	0.494	0.501	0.533	0.550	0.754
**Skilled Nursing facility admision**	AIC	13.1	12.5	12.5	12.3	12.1	10.8
	pR^2	7.5	11.9	11.7	13.4	14.9	24.0
	AUC ROC	0.692	0.753	0.749	0.768	0.782	0.849
	AUC_PR	0.177	0.235	0.236	0.258	0.274	0.388
**ER utilization (>2 visits)**	AIC	19.6	18.8	18.6	17.8	17.7	15.4
	pR^2	1.5	5.3	6.1	10.4	10.8	22.7
	AUC ROC	0.584	0.668	0.676	0.727	0.730	0.821
	AUC_PR	0.222	0.295	0.309	0.370	0.376	0.536
**PC utilization (> 21 visits)**	AIC	26.1	25.7	25.2	24.6	24.8	24.2
	pR^2	5.8	7.0	8.9	11.0	10.4	12.5
	AUC ROC	0.640	0.664	0.693	0.717	0.709	0.733
	AUC_PR	0.460	0.486	0.522	0.554	0.544	0.577
**Medications (>13 drugs)**	AIC	26.6	24.9	24.1	22.5	22.5	22.0
	pR^2	6.5	12.3	15.3	20.8	21.0	22.6
	AUC ROC	0.639	0.730	0.753	0.796	0.797	0.810
	AUC_PR	0.502	0.606	0.646	0.701	0.701	0.719

**Expenditure (>4,315.1 €)**	AIC	26.7	24.6	24.7	23.4	22.9	18.3
	pR^2	3.8	11.5	10.9	15.8	17.5	34.1
	AUC ROC	0.611	0.725	0.718	0.760	0.773	0.869
	AUC_PR	0.448	0.592	0.581	0.643	0.658	0.803

AUC_PR: area under the precision-recall. **AIC:** Akaike information criterion. **CCI:** Charlson Comorbidity Index. **ER:** emergency room. **GMA:** adjusted morbidity groups. **HCUP:** healthcare cost and utilization project of the US Agency for Healthcare Research and Quality. **PC:** primary care (>21 visits including general practitioner, nurse, and social worker, either at the primary care facility, home or via teleconsultation. **pR^2**: pseudo-R squared.

Cells are colored according to the goodness of fit of each model for a given statistic, from red (worse) to strong green (best).

Table S17: Performance of each model for multimorbidity measurement for explaining the selected outcomes, measured using four statistics. Chronic kidney disease

	Statistic	Age + Gender + socioeconomic status	+ CCI	+ QOF	+ Karolinska	+ HCUP	+ GMA
**All-cause death**	AIC	15.0	13.4	13.8	14.0	13.6	12.5
	pR^2	8.2	18.0	15.4	14.3	16.6	23.6
	AUC ROC	0.716	0.818	0.796	0.786	0.806	0.859
	AUC_PR	0.182	0.279	0.252	0.240	0.260	0.331
**Hospitalization**	AIC	34.9	32.2	32.2	31.0	30.4	25.0
	pR^2	1.1	8.6	8.7	12.2	13.8	29.2
	AUC ROC	0.569	0.703	0.698	0.733	0.748	0.842
	AUC_PR	0.359	0.509	0.511	0.554	0.572	0.741
**Non-scheduled hospitalization**	AIC	29.0	26.1	25.7	25.0	24.4	18.1
	pR^2	2.4	12.0	13.5	15.7	18.0	39.2
	AUC ROC	0.608	0.749	0.755	0.771	0.790	0.899
	AUC_PR	0.281	0.434	0.459	0.486	0.510	0.736
**Skilled Nursing facility admision**	AIC	14.1	13.0	13.0	12.7	12.4	10.8
	pR^2	6.2	13.7	14.0	15.6	17.9	28.1
	AUC ROC	0.689	0.786	0.787	0.800	0.819	0.882
	AUC_PR	0.132	0.210	0.219	0.236	0.256	0.375
**ER utilization (>2 visits)**	AIC	21.5	20.1	19.7	18.7	18.6	15.9
	pR^2	1.0	7.5	9.2	13.7	14.4	26.6
	AUC ROC	0.570	0.712	0.721	0.766	0.773	0.851
	AUC_PR	0.157	0.257	0.281	0.337	0.346	0.509
**PC utilization (> 21 visits)**	AIC	31.8	30.6	29.3	28.5	28.7	27.8
	pR^2	3.8	7.5	11.1	13.7	13.1	15.8
	AUC ROC	0.623	0.693	0.732	0.755	0.749	0.776
	AUC_PR	0.340	0.410	0.468	0.500	0.490	0.530
**Medications (>13 drugs)**	AIC	34.5	31.3	29.6	27.4	27.3	26.7
	pR^2	2.0	11.3	16.0	22.4	22.5	24.2
	AUC ROC	0.583	0.742	0.770	0.814	0.816	0.831
	AUC_PR	0.362	0.528	0.591	0.650	0.650	0.672

**Expenditure (>4,315.1 €)**	AIC	33.6	29.6	29.6	27.7	27.0	21.8
	pR^2	1.1	12.9	12.9	18.4	20.6	36.0
	AUC ROC	0.569	0.749	0.742	0.783	0.800	0.879
	AUC_PR	0.337	0.542	0.537	0.602	0.623	0.769

AUC_PR: area under the precision-recall. **AIC:** Akaike information criterion. **CCI:** Charlson Comorbidity Index. **ER:** emergency room. **GMA:** adjusted morbidity groups. **HCUP:** healthcare cost and utilization project of the US Agency for Healthcare Research and Quality. **PC:** primary care (>21 visits including general practitioner, nurse, and social worker, either at the primary care facility, home or via teleconsultation. **pR^2**: pseudo-R squared.

Cells are colored according to the goodness of fit of each model for a given statistic, from red (worse) to strong green (best).

Table S18: Performance of each model for multimorbidity measurement for explaining the selected outcomes, measured using four statistics. Chronic obstructive pulmonary disease

	Statistic	Age + Gender + socioeconomic status	+ CCI	+ QOF	+ Karolinska	+ HCUP	+ GMA
**All-cause death**	AIC	13.4	11.8	12.5	12.6	12.3	11.3
	pR^2	12.9	23.4	18.6	17.7	20.0	26.5
	AUC ROC	0.777	0.864	0.827	0.821	0.839	0.885
	AUC_PR	0.170	0.270	0.226	0.214	0.234	0.298
**Hospitalization**	AIC	39.7	37.2	37.3	35.9	35.6	30.2
	pR^2	4.8	10.8	10.6	13.9	14.7	27.5
	AUC ROC	0.647	0.725	0.720	0.751	0.758	0.837
	AUC_PR	0.369	0.490	0.487	0.526	0.539	0.695
**Non-scheduled hospitalization**	AIC	31.3	28.7	28.5	27.8	27.3	21.4
	pR^2	6.4	14.2	14.8	16.8	18.3	36.0
	AUC ROC	0.681	0.766	0.766	0.782	0.793	0.888
	AUC_PR	0.290	0.414	0.429	0.453	0.472	0.681
**Skilled Nursing facility admision**	AIC	12.3	11.3	11.4	11.2	10.9	9.7
	pR^2	12.8	20.2	19.5	20.8	22.8	31.7
	AUC ROC	0.780	0.847	0.838	0.847	0.861	0.909
	AUC_PR	0.136	0.207	0.211	0.225	0.243	0.348
**ER utilization (>2 visits)**	AIC	26.1	24.8	24.5	23.4	23.3	20.6
	pR^2	2.3	7.2	8.4	12.6	12.8	22.8
	AUC ROC	0.613	0.700	0.711	0.757	0.758	0.831
	AUC_PR	0.169	0.247	0.267	0.318	0.323	0.464
**PC utilization (> 21 visits)**	AIC	34.1	33.0	31.6	30.4	30.7	29.8
	pR^2	7.9	10.9	14.7	17.9	17.1	19.6
	AUC ROC	0.695	0.732	0.768	0.794	0.788	0.813
	AUC_PR	0.344	0.394	0.454	0.488	0.477	0.515
**Medications (>13 drugs)**	AIC	37.6	34.7	32.5	29.9	30.0	29.5
	pR^2	10.2	17.1	22.3	28.6	28.3	29.6
	AUC ROC	0.706	0.780	0.815	0.851	0.850	0.862
	AUC_PR	0.422	0.534	0.603	0.665	0.662	0.681

**Expenditure (>4,315.1 €)**	AIC	37.4	33.0	33.4	31.4	30.8	25.3
	pR^2	5.3	16.5	15.4	20.4	21.9	36.0
	AUC ROC	0.650	0.779	0.766	0.802	0.812	0.883
	AUC_PR	0.333	0.530	0.515	0.571	0.589	0.739

AUC_PR: area under the precision-recall. **AIC:** Akaike information criterion. **CCI:** Charlson Comorbidity Index. **ER:** emergency room. **GMA:** adjusted morbidity groups. **HCUP:** healthcare cost and utilization project of the US Agency for Healthcare Research and Quality. **PC:** primary care (>21 visits including general practitioner, nurse, and social worker, either at the primary care facility, home or via teleconsultation. **pR^2**: pseudo-R squared.

Cells are colored according to the goodness of fit of each model for a given statistic, from red (worse) to strong green (best).

Table S19: Performance of each model for multimorbidity measurement for explaining the selected outcomes, measured using four statistics. people aged >64 years and institutionalized in a nursing home for long-term care

	Statistic	Age + Gender + socioeconomic status	+ CCI	+ QOF	+ Karolinska	+ HCUP	+ GMA
**All-cause death**	AIC	6.7	6.4	6.5	6.5	6.5	6.3
	pR^2	2.7	5.8	5.3	4.5	4.8	8.2
	AUC ROC	0.614	0.670	0.662	0.650	0.654	0.702
	AUC_PR	0.276	0.329	0.320	0.308	0.312	0.363
**Hospitalization**	AIC	8.2	7.7	7.6	7.4	7.4	5.7
	pR^2	1.0	7.2	8.0	10.9	10.7	31.3
	AUC ROC	0.566	0.685	0.692	0.721	0.720	0.857
	AUC_PR	0.358	0.481	0.493	0.530	0.529	0.747
**Non-scheduled hospitalization**	AIC	7.8	7.3	7.2	7.0	7.0	5.2
	pR^2	1.0	7.7	8.9	11.3	11.3	34.0
	AUC ROC	0.567	0.695	0.703	0.727	0.727	0.874
	AUC_PR	0.319	0.446	0.464	0.496	0.499	0.738
**Skilled Nursing facility admision**	AIC	5.2	5.0	5.0	4.9	4.9	4.4
	pR^2	0.6	4.6	5.0	7.0	7.1	15.7
	AUC ROC	0.557	0.667	0.668	0.698	0.701	0.795
	AUC_PR	0.154	0.219	0.221	0.247	0.247	0.353
**ER utilization (>2 visits)**	AIC	4.9	4.6	4.6	4.4	4.4	3.7
	pR^2	1.3	6.9	8.3	12.1	11.8	26.5
	AUC ROC	0.581	0.702	0.711	0.753	0.750	0.853
	AUC_PR	0.153	0.237	0.259	0.304	0.302	0.492
**PC utilization (> 21 visits)**	AIC	6.1	6.0	5.9	5.7	5.8	5.6
	pR^2	0.9	3.3	4.5	7.2	6.3	9.6
	AUC ROC	0.567	0.637	0.655	0.696	0.685	0.731
	AUC_PR	0.201	0.250	0.271	0.303	0.290	0.339
**Medications (>13 drugs)**	AIC	8.6	8.0	7.8	7.4	7.4	7.3
	pR^2	0.8	7.2	10.5	14.3	14.1	16.2
	AUC ROC	0.558	0.687	0.716	0.750	0.750	0.768

	AUC_PR	0.380	0.517	0.564	0.606	0.605	0.634
**Expenditure (>4,315.1 €)**	AIC	7.5	7.0	6.9	6.6	6.6	5.2
	pR^2	1.7	8.8	9.8	14.1	13.8	31.9
	AUC ROC	0.590	0.705	0.713	0.752	0.751	0.863
	AUC_PR	0.315	0.447	0.458	0.513	0.511	0.708

AUC_PR: area under the precision-recall. **AIC:** Akaike information criterion. **CCI:** Charlson Comorbidity Index. **ER:** emergency room. **GMA:** adjusted morbidity groups. **HCUP:** healthcare cost and utilization project of the US Agency for Healthcare Research and Quality. **PC:** primary care (>21 visits including general practitioner, nurse, and social worker, either at the primary care facility, home or via teleconsultation. **pR^2**: pseudo-R squared.

Cells are colored according to the goodness of fit of each model for a given statistic, from red (worse) to strong green (best).

3.2 Supplementary Figures

Figure S2. Distribution of primary care visits in 10 subpopulations. The dotted line represent the 95th percentile in the general adult population (>17 years), and the percentage correspondes to individuals above this threshold (i.e., >21 visits yearly) in each sub-population.

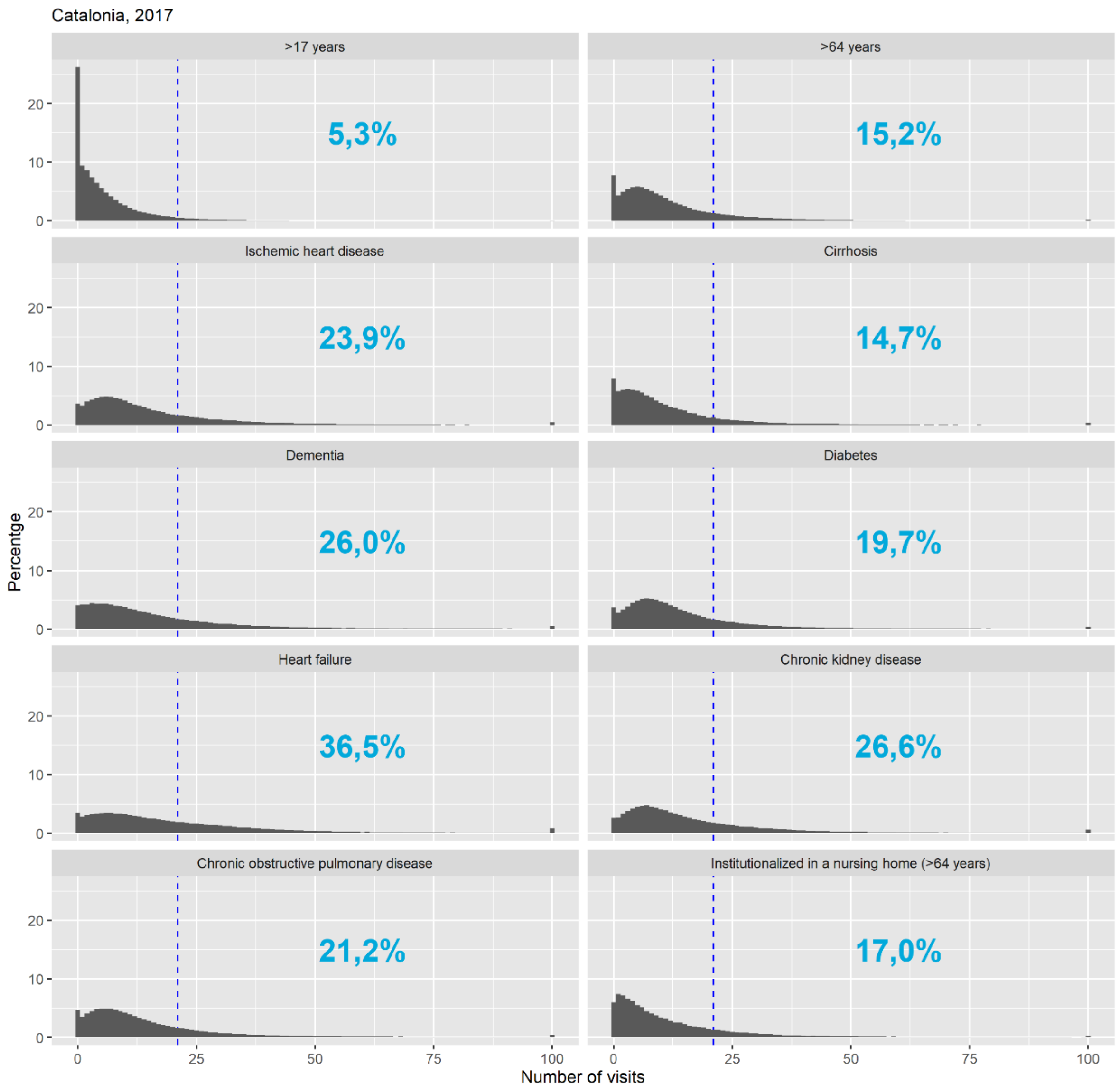

Figure S3. Distribution of health expenditure in 10 subpopulations. The dotted line represent the 95th percentile in the general adult population (>17 years), and the percentage correspondes to individuals above this threshold (i.e., > € 4315) in each sub-population.

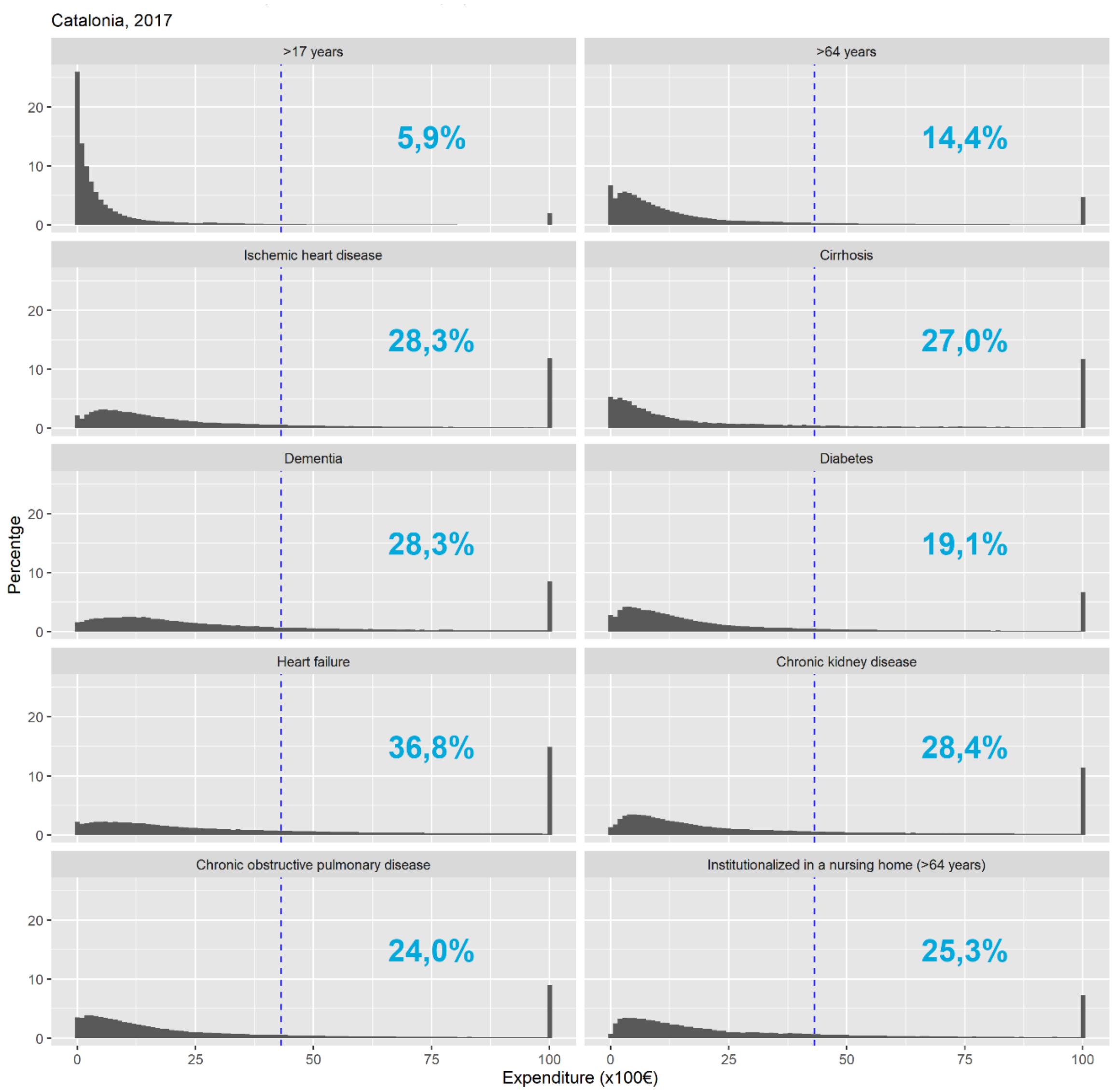

Figure S4. Distribution of medication use in 10 subpopulations. The dotted line represent the 95th percentile in the general adult population (>17 years), and the percentage correspondes to individuals above this threshold (i.e., > 13 drugs belonging to a different 5-digit group of the anatomic-therapeutic classification) in each sub-population.

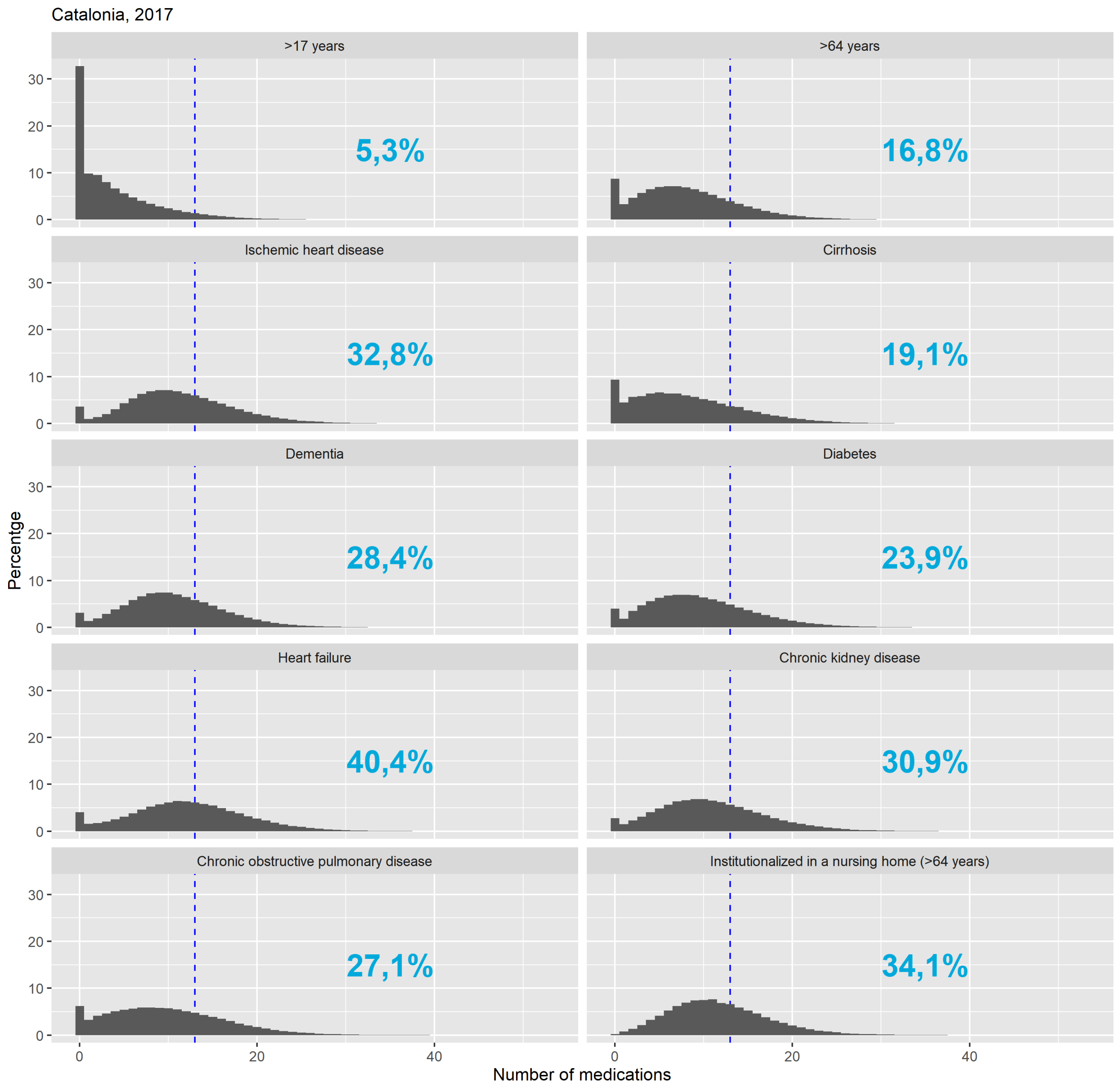

Figure S5. Distribution of emergency room visits in 10 subpopulations. The dotted line represent the 95th percentile in the general adult population (>17 years), and the percentage correspondes to individuals above this threshold (i.e., three or more admissions) in each sub-population.

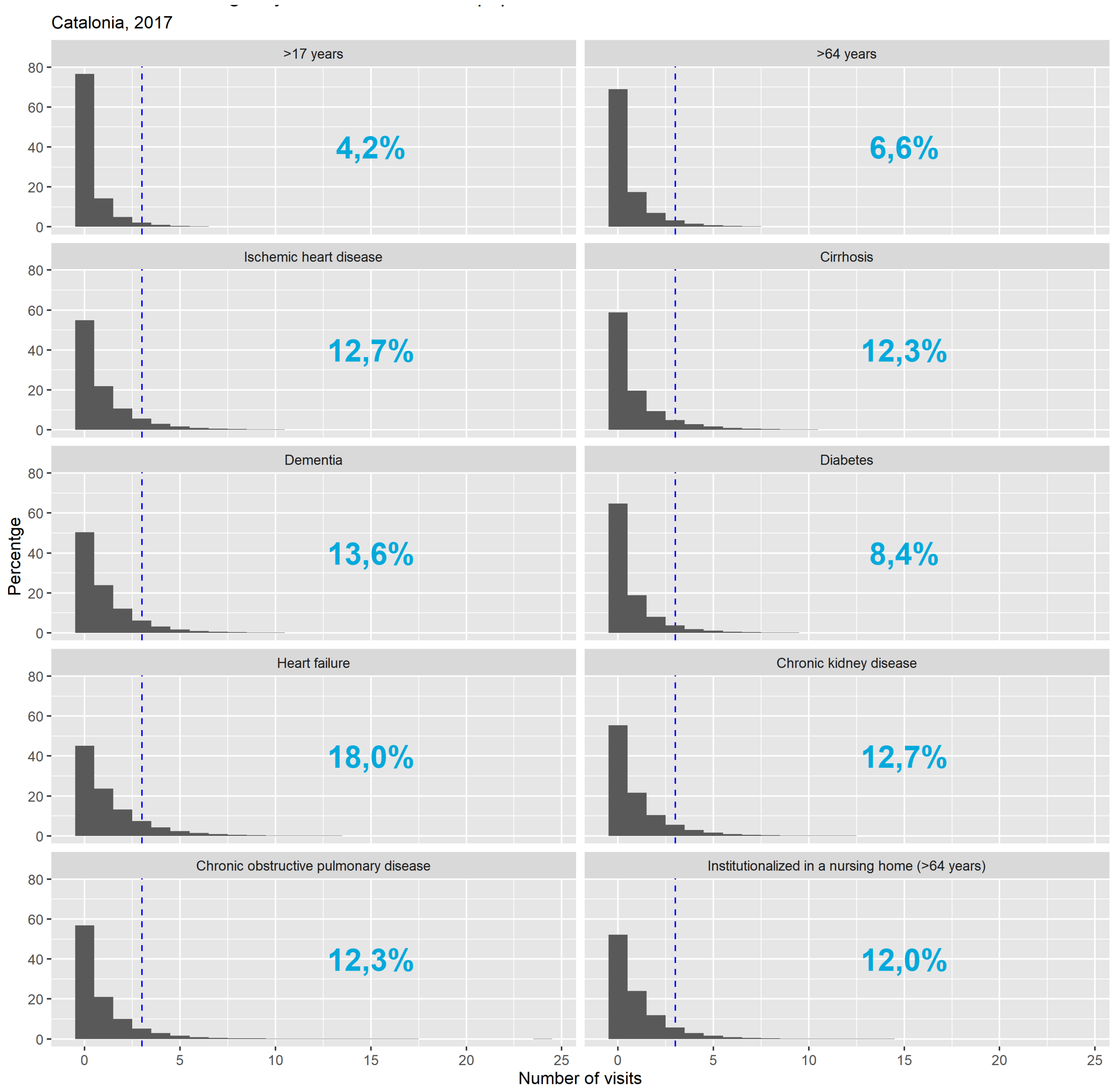

Supplement: Supplementary file 1 — Additional file 1: Supplementary methods (algorithm description) and supplementary results (Tables S1-S19 and Figures S1-S5). [file 12889_2021_11922_MOESM1_ESM.pdf]
